# Proportional Hazards Regression for Interval‐Censored Outcomes With an Interval‐Censored Covariate

**DOI:** 10.1002/sim.70573

**Published:** 2026-05-04

**Authors:** Dongdong Li, Yue Song, Wenbin Lu, Huldrych F. Günthard, Roger Kouyos, Rui Wang

**Affiliations:** ^1^ Department of Population Medicine Harvard Medical School and Harvard Pilgrim Health Care Institute Boston Massachusetts USA; ^2^ Department of Biostatistics Harvard T. H. Chan School of Public Health Boston Massachusetts USA; ^3^ Department of Statistics North Carolina State University Raleigh North Carolina USA; ^4^ Division of Infectious Diseases and Hospital Epidemiology University Hospital Zurich Zurich Switzerland; ^5^ Institute of Medical Virology, University of Zurich Zurich Switzerland

**Keywords:** composite likelihood, EM algorithm, HIV viral rebound, interval‐censored covariate

## Abstract

Identifying predictors for viral rebound trajectories after antiretroviral therapy (ART) interruption is central to HIV cure research. Motivated by the need to determine whether the time to achieve viral suppression after ART initiation can predict the time to viral rebound following ART interruption, we investigate modeling approaches that relate an interval‐censored outcome (e.g., time to viral rebound) and an interval‐censored covariate (e.g., time to viral suppression) under the assumption that viral load only crosses a threshold when bracketed by consecutive assessments. We develop estimation and inference procedures for fitting a proportional hazards regression model when both the outcome and a covariate are interval‐censored, without imposing parametric assumptions on the baseline hazard functions. To accommodate participants with multiple episodes of ART initiation and interruption, we extend the proposed method to account for the clustering of repeated observations within individuals. We derive the asymptotic properties of the proposed method and evaluate its finite‐sample performance through simulation studies. Applying the method to data from the Zurich Primary HIV Infection cohort, we find that a longer time to viral suppression during ART is associated with an increased hazard of viral rebound after ART interruption.

## 1 Introduction

Achieving sustained HIV remission without lifelong antiretroviral therapy (ART) is a central goal of HIV cure research. Upon ART interruption, individuals typically experience a rapid increase in viral load, followed by a decline and eventual stabilization at a post‐rebound set point. Understanding the dynamics of this viral rebound and identifying predictors of its key features are critical for developing and evaluating cure strategies.

One such viral rebound feature of interest is the *time to viral rebound*, defined as the time at which the viral load exceeds a clinically relevant threshold (e.g., 1000 copies/mL). Analytic treatment interruption (ATI) studies, in which participants are closely monitored following ART interruption, offer a unique opportunity to study rebound dynamics in a controlled setting. However, due to the intermittent timing of viral load measurements, the exact time of rebound is typically not observed but rather known to occur within an interval—resulting in interval‐censored data.

Evidence suggests that the HIV‐1 latent reservoir—a major barrier to viral remission—is largely established around the time of ART initiation [1]. Using non‐linear mixed effects models to jointly analyze viral decay following ART initiation and viral rebound after treatment interruption, Gao et al. [2] found that a faster rate of viral decay was associated with a lower viral setpoint upon rebound.

Our goal here is to identify predictors of *time to viral rebound* following ART interruption. In particular, we focus on the *time to viral suppression* after ART initiation, defined as the time to viral load dropping below the lower limit of assay quantification, as a potential predictor. As with time to rebound, time to suppression is also subject to interval censoring due to the periodic nature of viral load assessments.

In this paper, we consider proportional hazards regression models to relate an interval‐censored outcome and an interval‐censored covariate, without imposing parametric assumptions on the baseline hazard functions. While alternatives such as frailty or copula models can be used to assess the dependence between two event times, the regression framework offers a more direct evaluation of the predictive value of the covariate ($T_{1}$) on the outcome ($T_{2}$), with flexibility to adjust for additional covariates (e.g., age, sex).

Fitting Cox proportional hazards regression models with interval‐censored *outcomes* presents both theoretical and computational challenges, as estimating the regression coefficients cannot be easily separated from estimating the baseline hazard function. A variety of parametric, weakly parametric, or nonparametric methods have been proposed. For instance, Finkelstein [3] assumed a discrete baseline survival function and proposed to jointly estimate the baseline hazard function and regression coefficients using a Newton–Raphson algorithm. Pan [4] extended the iterative convex minorant algorithm to the Cox model with interval‐censored outcomes to overcome the computational challenges in inverting the Hessian matrix. Huang [5] proposed a maximum profile likelihood method and showed that the nonparametric maximum likelihood estimator for regression parameters is asymptotically normal with a $\sqrt{n}$ convergence rate, whereas the MLE for the infinite‐dimensional baseline cumulative hazard function converges at a slower rate.

Alternative approaches that avoid direct estimation of the baseline hazard include rank‐based and EM‐based methods. Satten [6] proposed a marginal likelihood approach, while Goggins et al. [7] developed a Monte Carlo Expectation–Maximization (EM) algorithm. Both approaches used the distribution of possible rankings of failure times consistent with the observed censoring intervals. See Wang et al. [8] and Zhang and Sun [9] for a comprehensive review of this topic. Zeng et al. [10] considered nonparametric maximum likelihood estimation for a broad class of semiparametric transformation models, including proportional hazards models. By augmenting the data with Poisson variables, they obtained a strictly concave reformulation of the likelihood, leading to stable EM algorithm convergence and asymptotically normal estimators. A similar Poisson augmentation technique was also used in Wang et al. [8] that employed splines for the baseline hazard function, and in multi‐state models with interval‐censored transition times [11, 12].

In contrast, the literature on interval‐censored *covariates* remains limited. Gómez et al. [13] considered a linear regression model with a continuous, fully observed outcome and an interval‐censored covariate, showing that midpoint imputation can be invalid and inefficient; they proposed a likelihood‐based two‐step conditional algorithm to jointly estimate the regression coefficients and the marginal distribution of the covariate. Langohr et al. [14] developed a parametric survival model relating a doubly‐censored survival outcome to an interval‐censored covariate, with estimation carried out via numerical optimization of the joint likelihood. Goggins et al. [15] proposed an EM algorithm to fit a Cox PH model with a right‐censored survival outcome and a binary time‐varying covariate whose change time is interval‐censored. Ahn et al. [16] jointly modeled a right‐censored survival outcome and an interval‐censored covariate, treating the latter as a partially observed time‐varying covariate. These contributions highlight that handling an interval‐censored covariate typically requires an auxiliary model for the covariate process and likelihood‐based inference rather than simple plug‐in (e.g., midpoint) approaches. However, each of these works assumes a specific outcome structure that differs from ours. Gómez et al. [13] study a fully observed continuous outcome in a linear regression framework, while Langohr et al. [14] assume a parametric survival model for a doubly‐censored outcome. In the semiparametric Cox‐model approaches of Goggins et al. [15] and Ahn et al. [16], the outcome is right‐censored, so the Cox likelihood for the outcome retains its standard form conditional on the covariate; the interval censoring of the covariate is then handled via EM or related imputation arguments. None of the above covers the setting where the outcome itself is also interval‐censored under a semiparametric model. Our motivating problem involves an interval‐censored outcome $T_{2}$ (which may also be right‐censored) together with an interval‐censored covariate $T_{1}$ (which may also be right‐censored), both arising from intermittent monitoring.

Fitting a proportional hazards model becomes particularly challenging when *both the outcome and a covariate* are interval‐censored, especially in the absence of parametric assumptions on the baseline hazard. To the best of our knowledge, this setting has not been previously addressed in either independent or clustered data contexts. Leveraging the Poisson augmentation technique [8, 10], we propose estimation and inference methods and a fast, computationally stable EM algorithm for fitting Cox proportional hazards models without making parametric distributional assumptions about the baseline hazard functions. We establish the asymptotic properties of the proposed method and evaluate its finite‐sample performances through simulation studies. Furthermore, we extend the method to handle clustered observations through the use of composite likelihood functions.

The remainder of this article is organized as follows. Section 2.1 introduces the notation, model, and the observed likelihood function in the independent data setting. Section 2.2 presents the proposed estimation and inference procedure, and Section 2.3 establishes its asymptotic properties. In Section 2.4, we extend the methods to accommodate clustered data. Section 3 presents results of simulation studies. In Section 4, we apply the methods to evaluate the effect of time to viral suppression on the hazard of viral rebound using data from the Zurich Primary HIV Infection Study (ZPHI). Finally, Section 5 concludes with some discussion and remarks.

## 2 Methods

### 2.1 Notation, Model and Observed Likelihood

Suppose we are interested in estimating the effect of the time to viral suppression (Event#1) after a patient starts ART on the time to viral rebound (Event#2) after stopping ART. We first consider the setting where there are N independent individuals. Let $\left(T_{1i},T_{2i}\right),i=1,\ldots ,N$ denote the event times for the i‐th individual for Event#1 and Event#2, respectively. Let ${\left\{U_{\mathrm{si}}^{\left(k\right)}\right\}}_{1:K_{\mathrm{si}}}$ denote the vector of temporally ordered monitoring times for $T_{\mathrm{si}}$, where $K_{\mathrm{si}}$ is the number of monitoring visits for the i‐th subject and Event#s, s=1,2, and $k=1,\ldots ,K_{\mathrm{si}}$. We define $U_{\mathrm{si}}^{\left(0\right)}=0$ and $U_{\mathrm{si}}^{\left(K_{\mathrm{si}}+1\right)}=\infty$. We then use $\left(L_{\mathrm{si}},R_{\mathrm{si}}\right]$ to denote the shortest interval in ${\left\{U_{\mathrm{si}}^{\left(k\right)}\right\}}_{0:\left(K_{\mathrm{si}}+1\right)}$ that brackets the true event time $T_{\mathrm{si}}$. When $T_{\mathrm{si}}$ has not yet occurred by the last assessment time $U_{\mathrm{si}}^{\left(K_{\mathrm{si}}\right)}$, the observation is right‐censored: the bracket becomes $\left(L_{\mathrm{si}},R_{\mathrm{si}}\right]=\left(U_{\mathrm{si}}^{\left(K_{\mathrm{si}}\right)},\infty \right)$, indicating that $T_{\mathrm{si}}$ is only known to exceed $U_{\mathrm{si}}^{\left(K_{\mathrm{si}}\right)}$. Let $Z_{\mathrm{si}}$ denote the random vector of covariates specific to Event#s for the i‐th subject. The observed covariate vectors for the i‐th subject are denoted by $z_{1i}$ and $z_{2i}$ for Event#1 and Event#2, respectively. The observed data is represented by $𝒟=\cup_{i=1}^{N}{𝒟}_{i}=\cup_{i=1}^{N}\left\{L_{1i},R_{1i},L_{2i},R_{2i},z_{1i},z_{2i}\right\}$. Finally, we assume that the monitoring schedule is noninformative. That is, the monitoring times ${\left\{U_{\mathrm{si}}^{\left(k\right)}\right\}}_{1:K_{\mathrm{si}}}$ are independent of $T_{1i}$ conditional on $Z_{1i}$ and also independent of $T_{2i}$ conditional on $\left(T_{1i},Z_{2i}\right)$. We further assume that the observed bracketing interval $\left(L_{\mathrm{si}},R_{\mathrm{si}}\right]$ correctly localizes the true event time $T_{\mathrm{si}}$. That is, the event of interest (i.e., the first threshold crossing for Event#s) does not occur and subsequently reverse between two consecutive assessment times unless those assessments bracket the event. In practice, this assumption is more plausible when the underlying trajectory is approximately monotone with respect to the relevant threshold during the phase of interest and/or when monitoring is sufficiently frequent to capture clinically meaningful threshold crossings between visits.

Let $\lambda_{\mathrm{si}}\left(t\right)$ represent the hazard function at time t for Event#s and the i‐th subject, and let $\lambda_{s}^{\left(0\right)}\left(t\right)$ represent the baseline hazard function for Event#s, s=1,2. The cumulative baseline hazard function for Event #s is denoted by $\Lambda_{s}^{\left(0\right)}\left(t\right)=\int_{0}^{t}\lambda_{s}^{\left(0\right)}\left(u\right)\mathrm{du}$. We model the outcome of interest, time to Event#2, using the following proportional hazards model:

(1) $$\lambda_{2i}\left(t\right)=\lambda_{2}^{\left(0\right)}\left(t\right)\exp\left(\beta_{1}T_{1i}+\beta_{2}^{⊤}Z_{2i}\right)$$

where $\beta_{1}$ is the primary parameter of interest, while $\beta_{2}$ represents the coefficient vector for the covariates $Z_{2i}$. For time to Event#1, we assume:

(2) $$\lambda_{1i}\left(t\right)=\lambda_{1}^{\left(0\right)}\left(t\right)\exp\left(\gamma^{⊤}Z_{1i}\right)$$

where the vector γ comprises coefficients for $Z_{1i}$. The covariate sets $Z_{1}$ and $Z_{2}$ may overlap partially, be completely distinct, or coincide entirely.

Based on model (1) and (2), the observed likelihood is given by:

(3) $$\begin{array}{ll} & {ℒ}_{\mathrm{obs}}^{*}\left(\beta_{1},\beta_{2},\gamma ,\Lambda_{1}^{\left(0\right)}\left(\cdot \right),\Lambda_{2}^{\left(0\right)}\left(\cdot \right);𝒟\right)\\ & =\prod_{i=1}^{N}\int_{L_{i1}}^{R_{i1}}\{\exp\left[-\exp\left(\beta_{1}t+\beta_{2}^{⊤}z_{2i}\right)\Lambda_{2}^{\left(0\right)}\left(L_{2i}\right)\right]\\ & \;-\exp\left[-\exp\left(\beta_{1}t+\beta_{2}^{⊤}z_{2i}\right)\Lambda_{2}^{\left(0\right)}\left(R_{2i}\right)\right]\}\\ & \;\times \exp\left[-\exp\left(\gamma^{⊤}z_{1i}\right)\Lambda_{1}^{\left(0\right)}\left(t\right)\right]\lambda_{1}^{\left(0\right)}\left(t\right)\exp\left(\gamma^{⊤}z_{1i}\right)\mathrm{dt} \end{array}$$

The above likelihood construction highlights why interval censoring of the covariate $T_{1}$ leads to a fundamentally different estimation problem from standard Cox regression with fully observed covariates. Because $T_{1}$ enters the hazard of $T_{2}$ through $\exp\left(\beta_{1}T_{1}\right)$ but is only observed through $\left(L_{1i},R_{1i}\right]$, inference for $\beta_{1}$ requires integrating over the latent $T_{1}$ distribution induced by the working model for $T_{1}$; consequently, the standard Cox partial likelihood is not available. Several previously studied settings arise as special cases of our framework. If $T_{1}$ were fully observed, our method reduces to semiparametric Cox regression for an interval‐censored outcome. If $T_{2}$ were right‐censored (rather than interval‐censored) while $T_{1}$ remained interval‐censored, the problem reduces to Cox regression with an interval‐censored covariate process, a setting studied in earlier work (e.g., Ahn et al. [16]). The distinguishing feature of the present work is that $T_{2}$ is interval‐censored (and may also be right‐censored) and $T_{1}$ is interval‐censored (and may also be right‐censored), requiring joint integration over uncertainty in both event times and nonparametric estimation of baseline hazard functions for both processes (Section 2.2.1); Poisson augmentation (Section 2.2.2) yields a computationally stable EM algorithm for likelihood‐based inference in this setting. Section 2.4 extends the method to clustered episode data via composite likelihood.

### 2.2 Estimation Procedure

#### 2.2.1 Nonparametric Estimation of Cumulative Baseline Hazard Functions

We estimate the cumulative baseline hazard functions of $T_{1}$ and $T_{2}$ nonparametrically as step functions. Specifically, let $0=t_{10}<t_{11}<\ldots <t_{1m_{1}}$ denote the ordered sequence consisting of 0 and all the unique finite values of the brackets $\left(L_{1i},R_{1i}\right]$, i=1,…,N, and similarly define $0=t_{20}<t_{21}<\ldots <t_{2m_{2}}$ based on $L_{2i}$ and $R_{2i}$. The estimates of $\Lambda_{1}^{\left(0\right)}\left(t\right)$, $\Lambda_{2}^{\left(0\right)}\left(t\right)$ are step functions with non‐negative jumps only at $t_{1\ell },l=1,\ldots ,m_{1}$, and $t_{2k},k=1,\ldots ,m_{2}$, respectively. At $t_{1\ell }$, the jump size of $\Lambda_{1}^{\left(0\right)}\left(\cdot \right)$ is denoted by $\lambda_{1\ell }$, and similarly, the jump size of $\Lambda_{2}^{\left(0\right)}\left(\cdot \right)$ at $t_{2k}$ is denoted by $\lambda_{2k}$. Additionally, we define $\lambda_{10}=\lambda_{20}=0$. The conditional distribution of $T_{1}$ is consequently discretized as $P\left(T_{1i}=t_{1\ell }|z_{1i}\right)=e^{-\exp\left(\gamma^{⊤}z_{1i}\right)\sum_{l^{\prime }\leq l-1}\lambda_{1l^{\prime }}}-e^{-\exp\left(\gamma^{⊤}z_{1i}\right)\sum_{l^{\prime }\leq l}\lambda_{1l^{\prime }}}=e^{-\exp\left(\gamma^{⊤}z_{1i}\right)\sum_{l^{\prime }\leq l-1}\lambda_{1l^{\prime }}}\left(1-e^{-\exp\left(\gamma^{⊤}z_{1i}\right)\lambda_{1l}}\right)$, for $l=1,\ldots ,m_{1}$.

Let $\theta ={\left(\beta_{1},\beta_{2}^{⊤},\gamma^{⊤},\lambda_{11},\ldots ,\lambda_{1m_{1}},\lambda_{21},\ldots ,\lambda_{2m_{2}}\right)}^{⊤}$ as the full parameter vector. We can then re‐express the likelihood of the observed data as:

(4) $$\begin{array}{ll} & {ℒ}_{\mathrm{obs}}\left(\theta ;𝒟\right)\\ & \;=\prod_{i=1}^{N}\sum_{L_{1i}<t_{1\ell }\leq R_{1i}}\left\{e^{-\exp\left(\beta_{1}t_{1\ell }+\beta_{2}^{⊤}z_{2i}\right)\sum_{t_{2k}\leq L_{2i}}\lambda_{2k}}-e^{-\exp\left(\beta_{1}t_{1\ell }+\beta_{2}^{⊤}z_{2i}\right)\sum_{t_{2k}\leq R_{2i}}\lambda_{2k}}\right\}\\ & \;\times e^{-\exp\left(\gamma^{⊤}z_{1i}\right)\sum_{l^{\prime }\leq l-1}\lambda_{1l^{\prime }}}\left(1-e^{-\exp\left(\gamma^{⊤}z_{1i}\right)\lambda_{1l}}\right) \end{array}$$

#### 2.2.2 Poisson Augmentation and EM Algorithm

The Poisson augmentation technique has been introduced by Zeng et al. [10] for maximum likelihood estimation under semiparametric regression models with interval‐censored data via the EM algorithm. The key idea is to adopt an equivalent representation of the observed data using latent Poisson random variables, which results in a convex optimization problem that admits closed‐form solutions for the baseline hazard increments $\lambda_{2k}$ in the maximization step. The methodology was developed under the setting where only the outcomes are interval‐censored. Building upon their work, we present an EM algorithm with Poisson augmentation for the scenario where both the outcome and a covariate are interval‐censored. The algorithm proceeds as follows. Full derivations and closed‐form expressions are given in Supporting Information Section A.

**Initialize**. Set starting values $\theta_{0}=(\beta_{1,0},\beta_{2,0},\gamma_{0},\lambda_{11,0},\ldots ,\lambda_{1m_{1},0},\lambda_{21,0},\ldots ,\lambda_{2m_{2},0})$.

**E‐step**. Given the current estimates $\theta_{r}$, compute:

- ${\hat{p}}_{i\ell }=E_{\theta_{r}}\left[I\left(T_{1i}=t_{1\ell }\right)|{𝒟}_{i}\right]$: the posterior probability that $T_{1i}$ equals the discretized support point $t_{1\ell }$, for each $t_{1\ell }\in \left(L_{1i},R_{1i}\right]$. This weight reflects the joint information from the $T_{1}$ model and the $T_{2}$ model.
- ${\hat{W}}_{\mathrm{ik}\ell }=E_{\theta_{r}}\left[W_{\mathrm{ik}}I_{i\ell }|{𝒟}_{i}\right]$: the conditional expectation of the latent Poisson count $W_{\mathrm{ik}}$ (at time grid point $t_{2k}$) weighted by the indicator that $T_{1i}=t_{1\ell }$, for each $t_{2k}\in \left(L_{2i},R_{2i}\right]$.

**M‐step**. Maximize the expected augmented log‐likelihood $𝒬\left(\theta |\theta_{r}\right)$ in two blocks:

- $T_{1}$ *parameters* $\left(\lambda_{11},\ldots ,\lambda_{1m_{1}},\gamma \right)$: solve the score equations for $\lambda_{1\ell }$ and γ via Newton–Raphson.
- $T_{2}$ *parameters* $\left(\lambda_{21},\ldots ,\lambda_{2m_{2}},\beta_{1},\beta_{2}\right)$: obtain ${\hat{\lambda }}_{2k}\left(\beta_{1},\beta_{2}\right)$ in closed form by profiling, then solve the remaining score equations for $\left(\beta_{1},\beta_{2}\right)$ via Newton–Raphson.

**Iterate**. Repeat the E‐step and M‐step until convergence. To estimate the variance, we compute the observed information matrix ${ℐ}_{\mathrm{obs}}\left(\theta \right)=-\partial^{2}\log{ℒ}_{\mathrm{obs}}\left(\theta ;𝒟\right)/\partial \theta \partial \theta^{⊤}$ evaluated at $\hat{\theta }$; the model‐based variance is ${ℐ}_{\mathrm{obs}}^{-1}\left(\hat{\theta }\right)$.

### 2.3 Asymptotic Properties

The following theorems establish the asymptotic properties of the proposed estimator $\left({\hat{\xi }}^{⊤},{\hat{\Lambda }}_{1},{\hat{\Lambda }}_{2}\right)$, where $\hat{\xi }={\left({\hat{\beta }}_{1},{\hat{\beta }}_{2}^{⊤},{\hat{\gamma }}^{⊤}\right)}^{⊤}$, and ${\hat{\Lambda }}_{1}$ and ${\hat{\Lambda }}_{2}$ are the estimators for the baseline hazard functions, $\Lambda_{1}$ and $\Lambda_{2}$, respectively. We denote the true parameter values as $\xi_{0}$, $\Lambda_{01}\left(\cdot \right)$, and $\Lambda_{02}\left(\cdot \right)$, respectively. The regularity conditions are provided in Supporting Information, Section C.1.

Theorem 1 *(Consistency). Suppose the regularity conditions (RC1–RC5) hold. Then, as* n→∞,

$$\hat{\xi }\overset{a.s.}{\to }\xi_{0}.$$

*Moreover, for any continuity points* τ *of* $\Lambda_{01}\left(\cdot \right)$ *and* υ *of* $\Lambda_{02}\left(\cdot \right)$, *we have*

$${\hat{\Lambda }}_{1}\left(\tau \right)\overset{a.s.}{\to }\Lambda_{01}\left(\tau \right),\;{\hat{\Lambda }}_{2}\left(\upsilon \right)\overset{a.s.}{\to }\Lambda_{02}\left(\upsilon \right).$$

*Thus, the proposed estimators for both the regression parameters and the baseline cumulative hazard functions are strongly consistent*.

Theorem 2 *(Rate of Convergence). Define the metric* d *on* ${\mathbb{R}}^{p}\times \Phi \times \Phi$ *as*

$$\begin{array}{cc} & d\left(\left(\xi_{1},\Lambda_{11},\Lambda_{21}\right),(\xi_{2},\Lambda_{12},\Lambda_{22})\right)\\ & \;=\;∥\xi_{1}-\xi_{2}∥+∥\Lambda_{11}-\Lambda_{12}{∥}_{2}+∥\Lambda_{21}-\Lambda_{22}{∥}_{2}, \end{array}$$

*where* Φ *is the space of cumulative hazard functions satisfying* 1/M≤Λ(τ)≤M *for some constant* M>0, ∥⋅∥ *denotes the Euclidean norm, and the* $L_{2}$ *‐norm of the difference between two cumulative hazard functions is given by*

$$∥\Lambda_{1}-\Lambda_{0}{∥}_{2}={\left[\int {\left(\Lambda_{1}\left(\nu \right)-\Lambda_{0}\left(\nu \right)\right)}^{2}\mathrm{dG}\left(\nu \right)\right]}^{1/2},$$

*where* G(ν) *is the marginal probability for monitoring time. Then, we have*

$$d\left(\left(\hat{\xi },{\hat{\Lambda }}_{1},{\hat{\Lambda }}_{2}\right),\left(\xi_{0},\Lambda_{01},\Lambda_{02}\right)\right)=O_{p}\left(n^{-1/3}\right).$$

*The rate of convergence of the proposed estimators is governed by the nonparametric estimation of the cumulative baseline hazard functions, which follows the well‐established cube‐root asymptotic rate* $O_{p}\left(n^{-1/3}\right)$ [5].

Theorem 3 *(Asymptotic Normality). Suppose that the regularity conditions (RC1)–(RC5) hold. Then, as* n→∞, *the estimator* $\hat{\xi }$ *satisfies*

$$\sqrt{n}\left(\hat{\xi }-\xi_{0}\right)\overset{d}{\to }N\left(0,\sum \right),$$

*where* ∑ *is the asymptotic variance–covariance matrix, which attains the semiparametric efficiency bound*.

The proofs generally follow the framework established in Zeng et al. [10], Gao et al. [17], and Huang [5]. An outline of proof is provided in the Supporting Information, Section C.2.

### 2.4 Extension to Clustered Data Settings

#### 2.4.1 Data and Model

Clustered survival data often emerge in clinical studies. In some cases, individuals may experience more than one episode of (Event#1, Event#2) pair. For instance, there were participants in the ZPHI who experienced multiple treatment interruptions, that is, the treatment status of a patient could be “on‐off‐on‐off‐…”. Consequently, for those participants, we observed more than one set of interval‐censored $\left(T_{1},T_{2}\right)$.

Let $n_{i}$ denote the number of episodes contributed by individual i, and $\left(T_{1\mathrm{ij}},T_{2\mathrm{ij}}\right)$ are the event times of episode j for individual i, i=1,…,N, $j=1,\ldots ,n_{i}$. Let ${\left\{U_{\mathrm{sij}}^{\left(k\right)}\right\}}_{1:K_{\mathrm{sij}}}$ denote the vector of temporally‐ordered monitoring times for $T_{\mathrm{sij}}$, s=1,2, where $K_{\mathrm{sij}}$ is the number of monitorings performed and $k=1,\ldots ,K_{\mathrm{sij}}$. We define $U_{\mathrm{sij}}^{\left(0\right)}=0$ and $U_{\mathrm{sij}}^{\left(K_{\mathrm{sij}}+1\right)}=\infty$. We use $\left(L_{\mathrm{sij}},R_{\mathrm{sij}}\right]$ to denote the shortest interval in ${\left\{U_{\mathrm{sij}}^{\left(k\right)}\right\}}_{0:\left(K_{\mathrm{sij}}+1\right)}$ that brackets $T_{\mathrm{sij}}$, and use $Z_{\mathrm{sij}}$ to denote the covariate vectors for individual i at the j‐th episode of Event#s.

In our data example, a considerable proportion of participants resumed ART after a period of treatment interruption and remained on ART until the end of follow‐up. In the clustered setting, an episode refers to a single ART‐interruption cycle indexed by j, during which we track the time to suppression $T_{1\mathrm{ij}}$ after ART initiation/re‐initiation and, when it occurs and is observed under the monitoring scheme, the subsequent time to rebound $T_{2\mathrm{ij}}$ after the next ART interruption. As a result, for these participants we only observed $\left(L_{1{\text{in}}_{i}},R_{1{\text{in}}_{i}}\right]$ but not $\left(L_{2{\text{in}}_{i}},R_{2{\text{in}}_{i}}\right]$ for their last episode. To account for this, we introduce the indicator $O_{2\mathrm{ij}}=1$ if $\left(L_{2\mathrm{ij}},R_{2\mathrm{ij}}\right]$ is observed and 0 otherwise, to accommodate partially observed data where only $T_{1}$ is available. Let ${𝒟}_{\mathrm{ij}}=\left\{L_{1\mathrm{ij}},R_{1\mathrm{ij}},O_{2\mathrm{ij}}L_{2\mathrm{ij}},O_{2\mathrm{ij}}R_{2\mathrm{ij}},z_{1\mathrm{ij}},z_{2\mathrm{ij}},O_{2\mathrm{ij}}\right\}$ denote the observed data of episode j from individual i, let ${𝒟}_{\mathrm{Ci}}=\cup_{j=1}^{n_{i}}{𝒟}_{\mathrm{ij}}$ denote the entire collection of observed clustered data contributed by individual i, and let ${𝒟}_{C}=\cup_{i=1}^{n}{𝒟}_{\mathrm{Ci}}$ denote the entire observed data. We assume that individuals are independent of one another, that is, ${𝒟}_{\mathrm{Ci}}⊥{𝒟}_{Ci^{\prime }}$ for $i\neq i^{\prime }$, but make no further assumptions about the correlation structure among observations contributed by the same individual. Finally, we assume that $n_{i}$, ${\left\{U_{\mathrm{sij}}^{\left(k\right)}\right\}}_{1:K_{\mathrm{sij}}}$ and $O_{2\mathrm{ij}}$ are independent of $T_{1\mathrm{ij}}$ conditioning on $Z_{1\mathrm{ij}}$ and also independent of $T_{2\mathrm{ij}}$ conditioning on $\left(T_{1\mathrm{ij}},Z_{2\mathrm{ij}}\right)$.

Similar to the independent data setting, the cumulative hazard functions will be estimated as step functions. The only difference is that the “jump points”, that is, $t_{1\ell }$ and $t_{2k}$, are now determined respectively by the entire collection of $\left\{L_{1\mathrm{ij}},R_{1\mathrm{ij}},i=1,\ldots ,N,j=1,\ldots n_{i}\right\}\setminus \left\{0,\infty \right\}$, and $\left\{O_{2\mathrm{ij}}L_{2\mathrm{ij}},O_{2\mathrm{ij}}R_{2\mathrm{ij}},i=1,\ldots ,N,j=1,\ldots n_{i}\right\}\setminus \left\{0,\infty \right\}$. A stratified analysis, where different $\Lambda_{1}^{\left(0\right)}\left(\cdot \right),\Lambda_{2}^{\left(0\right)}\left(\cdot \right)$ are assumed for each stratum, is also possible provided with reasonably large stratum sizes.

We adopt the composite likelihood approach and extend the proposed method to clustered data settings. An advantage of composite likelihood is that the estimation of regression coefficients can be carried out as if the observations were independent, which means that the algorithm can be applied to a clustered dataset with minimal alterations. Proper inference accounting for the clustering will be based on a bootstrap variance estimate or sandwich variance estimate instead of the model‐based variance estimate. Parallel to the independent data setting, the following Cox proportional hazards model for Event #2 is assumed:

(5) $$\lambda_{2\mathrm{ij}}\left(t\right)=\lambda_{2}^{\left(0\right)}\left(t\right)\exp\left(\beta_{1}T_{1\mathrm{ij}}+\beta_{2}^{⊤}Z_{2\mathrm{ij}}\right),$$

and for Event #1, the following model is assumed:

(6) $$\lambda_{1\mathrm{ij}}\left(t\right)=\lambda_{1}^{\left(0\right)}\left(t\right)\exp\left(\gamma^{⊤}Z_{1\mathrm{ij}}\right),$$

where $\beta_{1}$ is the primary parameter of interest, $\beta_{2}$ is the vector of covariate coefficients for $Z_{2\mathrm{ij}}$, and γ is the vector of covariate coefficients for $Z_{1\mathrm{ij}}$.

#### 2.4.2 Independence Composite Likelihood Function

The independence composite likelihood function is formulated by multiplying a collection of component likelihoods, whether or not they are independent [18, 19]. Specifically, we will base our inference on:

(7) $${ℒ}_{C}\left(\beta_{1},\beta_{2},\gamma ,\Lambda_{1}^{\left(0\right)}\left(\cdot \right),\Lambda_{2}^{\left(0\right)}\left(\cdot \right);{𝒟}_{C}\right)=\prod_{i=1}^{N}\prod_{j=1}^{n_{i}}f\left(\beta_{1},\beta_{2},\gamma ,\Lambda_{1}^{\left(0\right)}\left(\cdot \right),\Lambda_{2}^{\left(0\right)}\left(\cdot \right);{𝒟}_{\mathrm{ij}}\right)$$

where $f\left(\beta_{1},\beta_{2},\gamma ,\Lambda_{1}^{\left(0\right)}\left(\cdot \right),\Lambda_{2}^{\left(0\right)}\left(\cdot \right);{𝒟}_{\mathrm{ij}}\right)$ represents the likelihood contribution of episode j from individual i. The working independence assumption essentially reduces the dimension of the problem, and since each $f\left(\beta_{1},\beta_{2},\gamma ,\Lambda_{1}^{\left(0\right)}\left(\cdot \right),\Lambda_{2}^{\left(0\right)}\left(\cdot \right);{𝒟}_{\mathrm{ij}}\right)$ is a valid density, derivatives of the composite log‐likelihood still yield unbiased estimating equations. Solutions to the composite score equations are called the maximum composite likelihood estimators.

Under proper regularity conditions, they are shown to be consistent [19, 20, 21]. The composite likelihood approach results in valid estimators provided that the full‐data joint distributions are compatible with the component densities in ${ℒ}_{C}$, regardless of the structure of the higher‐order dependence among observations.

Using the notation for nonparametric estimation introduced in Section 2.2.1, the independent composite likelihood of the observed data in a clustered setting can be written as:

(8) $$\begin{array}{ll} & {ℒ}_{C}\left(\theta ;{𝒟}_{C}\right)\\ & =\prod_{i=1}^{N}\prod_{j=1}^{n_{i}}\sum_{L_{1\mathrm{ij}}<t_{1\ell }\leq R_{1\mathrm{ij}}}{\left\{e^{-\exp\left(\beta_{1}t_{1\ell }+\beta_{2}^{⊤}z_{2\mathrm{ij}}\right)\sum_{t_{2k}\leq L_{2\mathrm{ij}}}\lambda_{2k}}-e^{-\exp\left(\beta_{1}t_{1\ell }+\beta_{2}^{⊤}z_{2\mathrm{ij}}\right)\sum_{t_{2k}\leq R_{2\mathrm{ij}}}\lambda_{2k}}\right\}}^{O_{2\mathrm{ij}}}\\ & \;e^{-\exp\left(\gamma^{⊤}z_{1\mathrm{ij}}\right)\sum_{l^{\prime }\leq l-1}\lambda_{1l^{\prime }}}\left(1-e^{-\exp\left(\gamma^{⊤}z_{1\mathrm{ij}}\right)\lambda_{1l}}\right) \end{array}$$

The latent variables $W_{\mathrm{ijk}}$, $i=1,\ldots ,N,j=1,\ldots ,n_{i},k=1,\ldots ,m_{2}$, which, conditional on $\left(T_{1\mathrm{ij}},Z_{2\mathrm{ij}}\right)$, are independent Poisson random variables with means $\lambda_{2k}\exp\left(\beta_{1}T_{1\mathrm{ij}}+\beta_{2}^{⊤}Z_{2\mathrm{ij}}\right)$. In addition, let $I_{\mathrm{ij}\ell }$ denote the indicator $I\left(T_{1\mathrm{ij}}=t_{1\ell }|Z_{1\mathrm{ij}}\right)$. Define the augmented data collection for episode j of individual i by

$${𝒜}_{\mathrm{ij}}=\left\{I_{\mathrm{ij}\ell },z_{1\mathrm{ij}},z_{2\mathrm{ij}},O_{2\mathrm{ij}}W_{\mathrm{ijk}},O_{2\mathrm{ij}}:\ell =1,..,m_{1},k=1,\ldots ,m_{2}\right\}$$

The composite log‐likelihood for the augmented data ${𝒜}_{C}≜\cup_{i=1}^{n}\cup_{j=1}^{n_{i}}{𝒜}_{\mathrm{ij}}$ is, up to a constant,

$$\begin{array}{ll} & \log{ℒ}_{C}\left(\theta ;{𝒜}_{C}\right)\\ & =\sum_{i=1}^{N}\sum_{j=1}^{n_{i}}\sum_{L_{1\mathrm{ij}}<t_{1\ell }\leq R_{1\mathrm{ij}}}I_{\mathrm{ij}\ell }\left\{-\exp\left(\gamma^{⊤}z_{1\mathrm{ij}}\right)\sum_{l^{\prime }\leq l-1}\lambda_{1l^{\prime }}+\log\left(1-e^{-\exp\left(\gamma^{⊤}z_{1\mathrm{ij}}\right)\lambda_{1l}}\right)\right.\\ & \left.\;+\;O_{2\mathrm{ij}}\left[\sum_{t_{2k}\leq R_{2\mathrm{ij}}^{*}}W_{\mathrm{ijk}}\left(\log\lambda_{2k}+\beta_{1}t_{1\ell }+\beta_{2}^{⊤}z_{2\mathrm{ij}}\right)-\lambda_{2k}\exp\left(\beta_{1}t_{1\ell }+\beta_{2}^{⊤}z_{2\mathrm{ij}}\right)\right]\right\} \end{array}$$

The maximum composite likelihood estimators, denoted by $\hat{\theta }$, are derived using the EM algorithm with Poisson augmentation, as detailed in the Web Appendix B. The convergence properties of the composite likelihood EM algorithm have been established by Gao and Song [22].

Since the composite likelihood is constructed under a working independence assumption that ignores higher‐order correlations, it is the likelihood function of a misspecified model. Therefore, the model‐based variance is no longer valid. A sandwich variance estimator or a bootstrap approach can be used to compute the variance. The sandwich variance estimator is given by $A^{-1}{\mathrm{BA}}^{-1}$ where A can be estimated by the observed fisher information ${ℐ}_{\mathrm{obs}}\left(\hat{\theta }\right)$ as described in Section 2.2.2, and B is the estimated sum of $\left[\frac{\partial \log{ℒ}_{C}\left(\theta ;{𝒟}_{\mathrm{Ci}}\right)}{\partial \theta }\right]{\left[\frac{\partial \log{ℒ}_{C}\left(\theta ;{𝒟}_{\mathrm{Ci}}\right)}{\partial \theta }\right]}^{⊤}$ over all clusters i, where $\frac{\partial \log{ℒ}_{C}\left(\theta ;{𝒟}_{\mathrm{Ci}}\right)}{\partial \theta }$ can be estimated by evaluating the Jacobian of the observed log‐likelihood at the maximum composite likelihood estimator $\hat{\theta }$.

## 3 Simulation Studies

### 3.1 Independent Data Settings

#### 3.1.1 Study 1: Performance of the Proposed Method

The aim of this simulation study is to evaluate the performance of our proposed method in finite samples. We generated interval‐censored time‐to‐event data for N={100,200,400} individuals. The cumulative baseline hazard functions aligned with the Weibull distributions, specifically $\Lambda_{1}^{\left(0\right)}\left(t\right)={\left(t/3\right)}^{2}$ and $\Lambda_{2}^{\left(0\right)}\left(t\right)={\left(t/10\right)}^{5}$, and we considered the following model for the outcome event of interest:

(9) $$\lambda_{2i}\left(t\right)=\lambda_{20}\left(t\right)\exp\left(\beta_{1}T_{1i}+\beta_{2}Z_{i}\right),i=1,\ldots ,N$$

and the following model for Event#1:

(10) $$\lambda_{1i}\left(t\right)=\lambda_{10}\left(t\right)\exp\left(\gamma Z_{i}\right),i=1,\ldots ,N$$

where $Z_{i}∼\text{Bern}\;\left(0.5\right)$ is a binary covariate, and $\left(\beta_{1},\beta_{2},\gamma \right)=\left(0.3,1.0,-0.5\right)$. A binary covariate $Z_{i}$ was generated to reflect the motivating example, where the effect of sex on time to rebound was of interest. Additional simulation settings with continuous covariates were also considered, and the performance of the methods was similar to the settings presented here. Detailed results are provided in the Appendix D.1. For each $\left(T_{1i},T_{2i}\right)$ episode, we generated the monitoring schedule as follows:

$$\begin{array}{l} U_{1i}^{\left(1\right)}∼\text{Unif}\;\left(0,d_{1}\right),\;U_{1i}^{\left(h\right)}=U_{1i}^{\left(h-1\right)}+c_{1}+\text{Unif}\;\left(0,d_{1}\right),h=2,\ldots ,K_{1}\\ U_{2i}^{\left(1\right)}∼\text{Unif}\;\left(0,d_{2}\right),\;U_{2i}^{\left(h\right)}=U_{2i}^{\left(h-1\right)}+c_{2}+\text{Unif}\;\left(0,d_{2}\right),h=2,\ldots ,K_{2} \end{array}$$

To investigate the impact of monitoring frequency of either event, we simulated monitoring schedule of the event from combinations of minimum gap between monitoring times ($c_{s},s=1,2$), average gap (controlled by $d_{s}$), and the total number of monitoring ($K_{s}$). We defined two monitoring schedules as level 1 (low frequency: $c_{s}=1/15,d_{s}=1.0,K_{s}=30$), and level 2 (high frequency: $c_{s}=19/590,d_{s}=0.5,K_{s}=60$). We alternated the monitoring frequencies for the two events at each of the two levels, deriving four scenarios indexed by Scenario a.b, where a=1 or 2 and b=1 or 2, for the scenario when $T_{1}$ was monitored at level a and $T_{2}$ at level b. The starting values for the regression parameters $\left(\beta_{1},\beta_{2},\gamma \right)$ were set at zeros. Alternatively, one could use a naïve approach (e.g., use imputed times by the midpoint of the observed intervals and fit a Cox regression model) and obtain the point estimates for $\left(\beta_{1},\beta_{2},\gamma \right)$ as the starting values for potentially faster convergence. The starting values for $\lambda_{1\ell }$ and $\lambda_{2k}$ were set to be $1/m_{1}$ and $1/m_{2}$, respectively.

The parameters of interest were the regression coefficients $\left(\beta_{1},\beta_{2},\gamma \right)$. We fitted the models as specified in (1) and (2) and followed inference procedure in the Methods section to evaluate the performance of the proposed methods. We evaluated the performance of the proposed method by the bias, defined as the difference between the average of the estimates and true value, empirical standard error, the average of the standard error estimates, and the coverage probability of the confidence intervals for the regression coefficients.

Table 1 summarizes the results for sample sizes 100, 200, and 400 across 10000 replicates. The means of the estimates for the regression coefficients were close to the true values. The variance estimates were accurate, and the empirical coverage probabilities of confidence intervals using normal approximation were close to the 95% nominal level. Similar findings were observed across the four levels of combinations of monitoring frequency.

**TABLE 1:** Simulation results for the independent data setting (Study 1).

Sample size		BIAS	ESE	ASE	CP	BIAS	ESE	ASE	CP
		Scenario 1.1				Scenario 1.2			
n=100	$\beta_{1}=0.3$	0.005	0.075	0.072	0.943	0.006	0.073	0.071	0.946
	$\beta_{2}=1.0$	0.042	0.242	0.237	0.947	0.045	0.243	0.234	0.946
	γ=−0.5	−0.020	0.221	0.213	0.945	−0.017	0.218	0.213	0.945
n=200	$\beta_{1}=0.3$	−0.009	0.153	0.149	0.946	−0.010	0.149	0.149	0.953
	$\beta_{2}=1.0$	0.023	0.166	0.165	0.949	0.022	0.166	0.162	0.945
	γ=−0.5	−0.001	0.051	0.049	0.944	−0.001	0.050	0.049	0.946
n=400	$\beta_{1}=0.3$	−0.005	0.106	0.104	0.947	−0.006	0.106	0.105	0.948
	$\beta_{2}=1.0$	0.011	0.116	0.115	0.947	0.012	0.115	0.114	0.948
	γ=−0.5	−0.005	0.035	0.034	0.945	−0.005	0.035	0.034	0.940

		Scenario 2.1				Scenario 2.2			
n=100	$\beta_{1}=0.3$	0.011	0.074	0.072	0.946	0.012	0.074	0.071	0.942
	$\beta_{2}=1.0$	0.040	0.248	0.237	0.942	0.039	0.243	0.234	0.944
	γ=−0.5	−0.017	0.220	0.211	0.939	−0.017	0.220	0.211	0.943
n=200	$\beta_{1}=0.3$	0.005	0.051	0.050	0.945	0.005	0.050	0.049	0.945
	$\beta_{2}=1.0$	0.023	0.166	0.165	0.950	0.023	0.164	0.163	0.947
	γ=−0.5	−0.008	0.151	0.148	0.948	−0.009	0.148	0.148	0.950
n=400	$\beta_{1}=0.3$	0.001	0.035	0.035	0.950	0.002	0.035	0.034	0.945
	$\beta_{2}=1.0$	0.010	0.116	0.115	0.950	0.010	0.114	0.114	0.951
	γ=−0.5	−0.006	0.106	0.104	0.946	−0.005	0.106	0.104	0.945

*Note:* Number of replications 10 000.

Abbreviations: ASE, average standard error; CP, coverage probability; ESE, empirical standard deviation.

#### 3.1.2 Study 2: Comparison With Midpoint Imputation

The aim of this simulation study is to evaluate the performance of the proposed approach compared to methods using midpoint imputation. We generated $T_{2}$ following model (9) with $\left(\beta_{1},\beta_{2}\right)=\left(0.3,1.0\right)$. We generated $T_{1}$ following the model (10) with γ=0, which means that $T_{1}$ follows a Weibull distribution. The cumulative baseline hazard functions were specified as Weibull distributions with $\Lambda_{1}^{\left(0\right)}\left(t\right)={\left(t/15\right)}^{2}$ and $\Lambda_{2}^{\left(0\right)}\left(t\right)={\left(t/10\right)}^{2}$. Monitoring frequencies were generated to achieve two levels of monitoring frequency for $T_{1}$ and $T_{2}$, respectively, indexed by Scenario a.b as described in the above Simulation Study 1, for a=1,2, b=1,2. In addition, because follow‐up is administratively ended, both $T_{1}$ and $T_{2}$ can be right‐censored in our simulations. When $T_{s}$ is right‐censored we impute $T_{s}^{*}=L_{s}$ (since a midpoint is not defined for $\left(L_{s},\infty \right)$, s=1,2). The empirical right‐censoring rates for $T_{1}$ were similar across Scenarios 1.1, 1.2, 2.1, and 2.2, at approximately 28.4% in all four settings. The empirical right‐censoring rates for $T_{2}$ were also similar across the four scenarios and were all below 0.1% (see Table S2).

The parameters of interest were the regression coefficients $\left(\beta_{1},\beta_{2}\right)$. We carried out two midpoint‐imputed analyses by imputing the event times using the midpoint of their observed intervals. In midpoint‐imputed analysis (a), only $T_{1}$ was imputed, followed by fitting an interval‐censored Cox regression model [3] and variance estimation through bootstrapping. In midpoint‐imputed analysis (b), both $T_{1}$ and $T_{2}$ were imputed, and a Cox proportional hazards regression model was fitted. We compared the bias, standard error estimates, and coverage probability of the confidence intervals for the estimators of the regression coefficients.

**FIGURE 1:**
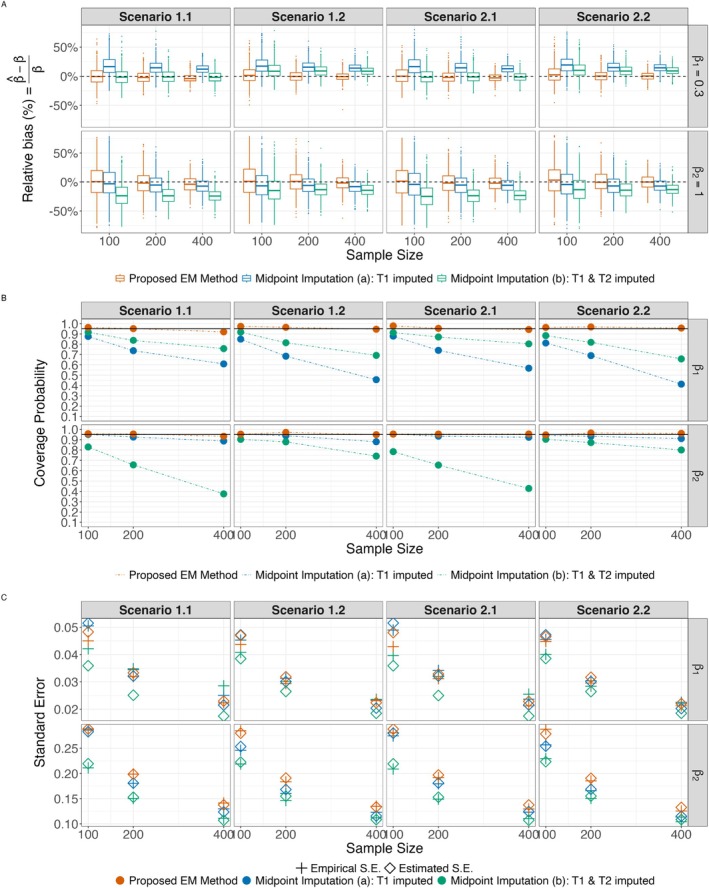
Simulation results comparing proposed method to two imputation methods (Study 2). Midpoint Imputation Method a: Only $T_{1}$ was imputed. Midpoint Imputation Method b: Both $T_{1}$ and $T_{2}$ were imputed. (A) Relative bias (%) of estimates. (B) Coverage probability of the interval estimate. (C) Mean standard error (SE) estimates versus the empirical SE. Number of replications 1000.

Figure 1 visualizes the comparison results across 1000 replicates. The two approaches using midpoint imputation were suboptimal, exhibiting bias (Panel A, Figure 1) and lower‐than‐nominal coverage (Panel B, Figure 1). For the proposed method, the standard error estimates were close to the empirical standard errors across all scenarios, whereas the midpoint‐imputed analyses underestimated the standard errors, particularly when the monitoring frequency was low (Panel C, Figure 1). In midpoint‐imputed analysis (a), where only $T_{1}$ was imputed, the coverage probability for $\beta_{1}$, the primary parameter of interest, decreased as the sample size increased. In midpoint‐imputed analysis (b), where both $T_{1}$ and $T_{2}$ were imputed, bias was observed in the estimates of both $\beta_{1}$ and $\beta_{2}$. For a given sample size, the observed bias in $\beta_{1}$ estimates increased with a higher monitoring frequency of $T_{2}$. This counterintuitive pattern arises from the partial cancellation of two opposing bias components in approach (b). Imputing $T_{1}$ introduces a positive bias in ${\hat{\beta }}_{1}$, largely due to right‐censoring of $T_{1}$ (approximately 28% of observations have $R_{1}=\infty$, where we impute $T_{1}^{*}=L_{1}$), systematically underestimating the covariate value. Since $T_{1}$ monitoring is identical in Scenarios 1.1 and 1.2, this positive bias component remains essentially constant. Imputing $T_{2}$ introduces a negative bias in ${\hat{\beta }}_{1}$ because coarser discretization of the outcome disrupts the event ordering in the Cox partial likelihood, attenuating the estimated covariate effect. This negative bias is larger in magnitude when $T_{2}$ intervals are wider (Scenario 1.1) and smaller when they are narrower (Scenario 1.2). Under approach (b), both imputation errors act simultaneously and their effects on ${\hat{\beta }}_{1}$ partially cancel. In Scenario 1.1 (wider $T_{2}$ intervals), the larger negative bias from $T_{2}$ imputation more effectively offsets the positive bias from $T_{1}$ imputation, resulting in a smaller net bias for ${\hat{\beta }}_{1}$. In Scenario 1.2 (narrower $T_{2}$ intervals), the $T_{2}$ imputation bias is smaller in magnitude, so less cancellation occurs and the net ${\hat{\beta }}_{1}$ bias is larger. This partial cancellation of opposing bias components explains the non‐monotone pattern: more frequent $T_{2}$ monitoring reduces $T_{2}$ imputation error but simultaneously removes the offsetting effect on the $T_{1}$ imputation bias, leading to a larger overall bias for ${\hat{\beta }}_{1}$. In contrast, with the same simulated data, the proposed approach consistently produced estimates close to the true values and maintained confidence interval coverage probabilities near 95%.

#### 3.1.3 Study 3: Robustness to Model Misspecification

The aim of this simulation study is to evaluate the robustness of the proposed method under model misspecification for $T_{1}$ and/or $T_{2}$.

**Study 3A: Robustness to covariate‐set misspecification**.

Data were generated following the same process as in Study 1, with a sample size of n=200. Additionally, we introduced an auxiliary covariate X, drawn from a standard normal distribution, to serve as a noise covariate. We considered two monitoring frequencies: Level 1 (low) and Level 2 (high).

Model misspecification was introduced through two mechanisms: (a) Omitting the true covariate Z from the model(s); (b) Including an additional noise covariate X in the model(s). We considered four model specifications for $T_{1}$ and $T_{2}$: (1) Benchmark (Correct Specification): Both models correctly included Z and excluded X. (2) Misspecified $T_{1}$, Correct $T_{2}$: The model for $T_{1}$ was misspecified (either omitting Z or including X), while the model for $T_{2}$ was correctly specified. (3) Correct $T_{1}$, Misspecified $T_{2}$: The model for $T_{2}$ was misspecified (either omitting Z or including X), while the model for $T_{1}$ was correctly specified. (4) Misspecified $T_{1}$ and $T_{2}$: Both models were misspecified (either omitting Z or including X).

We examined a total of 2 (monitoring frequency) × 2 (removing or adding covariates) × 4 (model specification combinations) = 16 scenarios. The parameter of interest was $\beta_{1}$. We assessed the impact of model misspecification by evaluating the bias, the coverage probability of the confidence intervals for the estimator of $\beta_{1}$, and the standard error estimates compared to empirical standard error.

**FIGURE 2:**
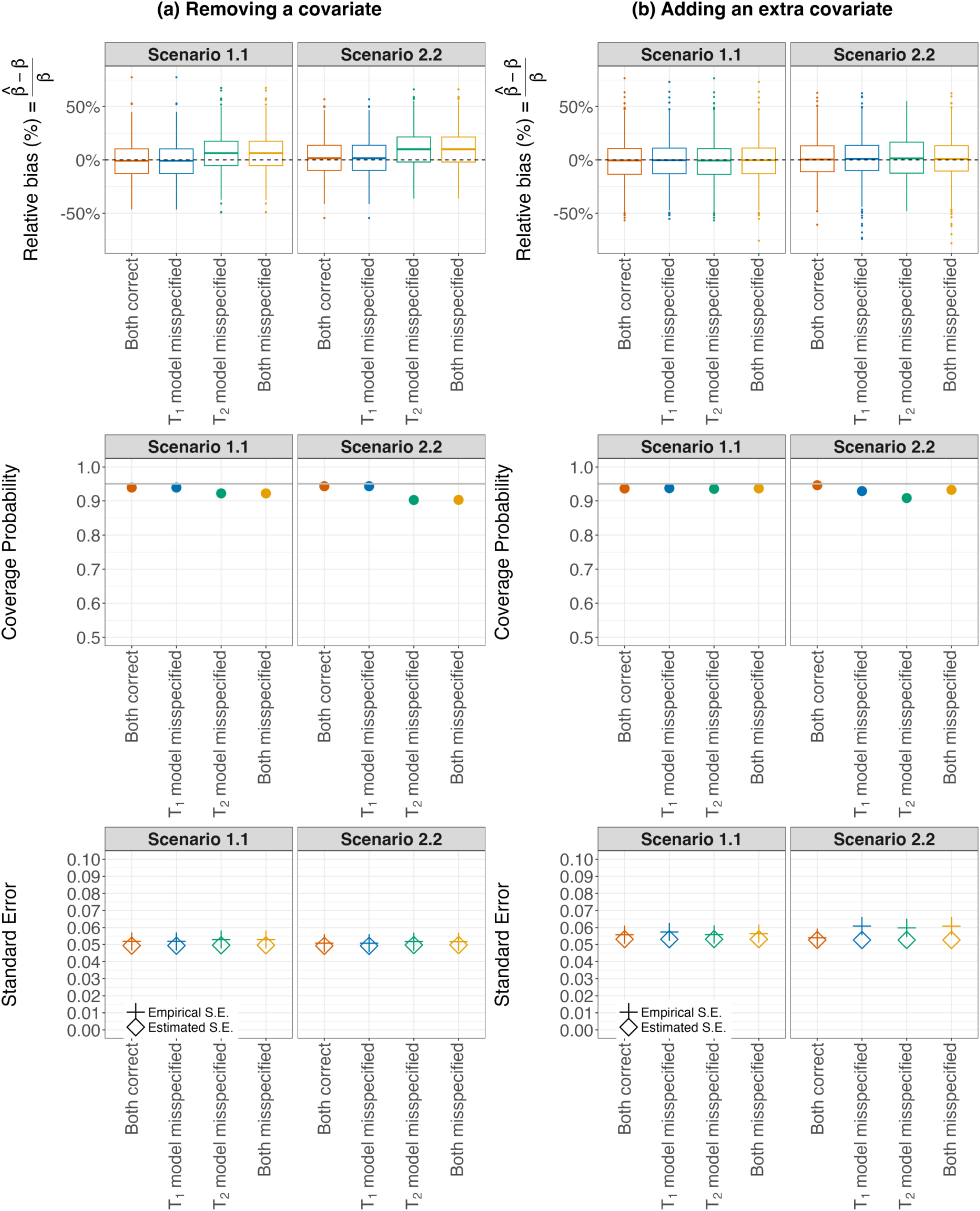
Simulation results for robustness study under model misspecification (Study 3A).

Figure 2 summarizes the results across 1000 replications. For the settings we examined, the proposed method generally demonstrated robustness to two forms of model misspecification: (a) omitting a relevant covariate (left panel, Figure 2) and (b) adding an irrelevant noise covariate X (right panel, Figure 2). Relative bias was modest across scenarios, especially when only the model for $T_{1}$ was misspecified or when the misspecification involved the inclusion of an extra covariate. Coverage probabilities were generally close to the nominal 95% level, with mild under‐coverage observed when the model for $T_{2}$ was misspecified, particularly under low‐frequency monitoring (Scenario 1.1). This under‐coverage improved with more frequent monitoring (Scenario 2.2). Estimated standard errors were generally consistent with empirical standard errors. As expected, misspecification had a greater impact when relevant covariates were omitted than when irrelevant covariates were included, as the latter case still maintained correct model specification aside from the redundant predictor. The impact of model misspecification is likely to be more pronounced when the omitted covariate has a stronger effect or when a large number of redundant covariates are included in the model.

**Study 3B: Robustness to violation of the proportional hazards assumption for** $T_{1}$.

We additionally evaluated robustness when the proportional hazards assumption for the first event time $T_{1}$ is violated. In this study, we kept the data‐generating mechanism for $T_{2}|\left(T_{1},Z_{2}\right)$, the baseline hazards, and the monitoring/interval‐censoring mechanism identical to those in Study 1, and modified only the hazard of $T_{1}$ to introduce a time‐varying covariate effect. Specifically, we generated $T_{1i}$ from the piecewise proportional hazards model

$$\lambda_{1i}\left(t|Z_{i}\right)=\lambda_{10}\left(t\right)\exp\left\{\gamma^{\left(1\right)}Z_{i}\;I\left(t\leq \tau \right)+\gamma^{\left(2\right)}Z_{i}\;I\left(t>\tau \right)\right\},$$

with $\gamma^{\left(1\right)}=\gamma$ and $\gamma^{\left(2\right)}=0$, where τ was set to the marginal median of $T_{1}$ under Z=0 in the corresponding Study 1 scenario. For estimation and inference, we intentionally fit the working PH model $\lambda_{1i}\left(t|Z_{i}\right)=\lambda_{10}\left(t\right)\exp\left(\gamma Z_{i}\right)$ together with the original working model for $T_{2}|\left(T_{1},Z_{2}\right)$. Following the reviewer's suggestion, we also included a comparator based on midpoint imputation for $T_{1}$ only (approach (a)) under the same data‐generating mechanism. The results, summarized in Table S3, show that the proposed approach retained negligible bias and near‐nominal coverage for $\beta_{1}$ in both the low‐ and high‐frequency monitoring scenarios (bias =−0.015 and 0.004; coverage =0.921 and 0.945), whereas midpoint imputation approach (a) exhibited larger positive bias and lower coverage (bias =0.046 and 0.058; coverage =0.896 and 0.882), under the settings we considered.

### 3.2 Clustered Data Settings

We performed additional simulation studies to evaluate the performance of the proposed method under clustered data settings, which are common in clinical studies where individuals may contribute multiple observations of $\left(L_{1},R_{1},L_{2},R_{2}\right)$, as in the case of HIV viral suppression‐rebound scenarios. To simulate this, we sampled the number of episodes $n_{i}$ from a discrete distribution with the probability of having {1,2,3} episodes being {0.3,0.5,0.2} respectively. As observations contributed by the same individual may be correlated, we first used a Gaussian copula with an exchangeable correlation matrix (off‐diagonal entries = 0.3, 0.5, 0.7 to represent different strengths of association) to generate the marginal probabilities $\left(s_{1i1},s_{1i2},\ldots ,s_{1{\text{in}}_{i}}\right)$, and then $\left(T_{1i1},T_{1i2},\ldots ,T_{1{\text{in}}_{i}}\right)$ were calculated according to the marginal model:

$$\lambda_{1\mathrm{ij}}\left(t\right)=\lambda_{1}^{\left(0\right)}\left(t\right)\exp\left(\gamma Z_{1\mathrm{ij}}\right),i=1,\ldots ,N,j=1,\ldots ,n_{i}$$

The same approach was used to generate $\left(T_{2i1},T_{2i2},\ldots ,T_{2{\text{in}}_{i}}\right)$, following the model

$$\lambda_{2\mathrm{ij}}\left(t\right)=\lambda_{2}^{\left(0\right)}\left(t\right)\exp\left(\beta_{1}T_{1\mathrm{ij}}+\beta_{2}Z_{2\mathrm{ij}}\right),i=1,\ldots ,N,j=1,\ldots ,n_{i}$$

where $Z_{\mathrm{ij}}∼\text{Bern}\left(0.5\right)$ was a binary covariate and $\left(\beta_{1},\beta_{2},\gamma \right)=\left(0.3,1.0,-0.5\right)$. The cumulative baseline hazard functions were $\Lambda_{1}^{\left(0\right)}\left(t\right)={\left(t/3\right)}^{2}$ and $\Lambda_{2}^{\left(0\right)}\left(t\right)={\left(t/10\right)}^{5}$.

For each individual's last episode, there was a 70% probability that $\left(L_{2},R_{2}\right)$ was unobserved, indicating that the patient resumed ART and did not experience another event until the end of the follow‐up. Thus, we only observed the interval‐censored time to viral suppression but not the subsequent time to rebound. For each $\left(T_{1\mathrm{ij}},T_{2\mathrm{ij}}\right)$ episode, we generated the monitoring schedules using the same scheme as in the independent data setting.

The parameters of interest were the regression coefficients $\left(\beta_{1},\beta_{2},\gamma \right)$. We fitted the models as specified in (5) and (6) and followed the inference procedure in the Methods section to evaluate the performance of the proposed methods. We evaluated the performance of the proposed methods under the settings described above. The performance metrics were the same as those in the independent data setting.

The results across 10 000 replications for ρ=0.5 are summarized in Table 2. The average point estimates for the regression coefficients were close to the true value. The sandwich variance estimator performed well, and the empirical coverage probabilities of confidence intervals based on the normal approximation were close to the nominal level. To assess sensitivity to the strength of within‐subject dependence, we repeated the clustered simulation under ρ∈{0.3,0.7}, with all other data‐generating components unchanged. The additional results (Table S4) for Scenarios 1.1 and 2.2 show that point estimation remains essentially unbiased across these dependence levels; the estimated standard errors and confidence‐interval coverage remain close to their empirical and nominal targets, respectively, consistent with the ρ=0.5 results above.

**TABLE 2:** Simulation results for the clustered data setting.

Sample size		BIAS	ESE	sASE	CP	BIAS	ESE	sASE	CP
		Scenario 1.1				Scenario 1.2			
n=100	$\beta_{1}=0.3$	0.000	0.057	0.054	0.928	0.004	0.057	0.053	0.932
	$\beta_{2}=1.0$	0.024	0.209	0.199	0.938	0.026	0.208	0.197	0.935
	γ=−0.5	−0.009	0.192	0.182	0.937	−0.007	0.193	0.182	0.941
n=200	$\beta_{1}=0.3$	−0.003	0.040	0.038	0.935	−0.002	0.040	0.038	0.933
	$\beta_{2}=1.0$	0.013	0.144	0.141	0.942	0.017	0.144	0.140	0.942
	γ=−0.5	−0.005	0.135	0.130	0.940	−0.006	0.134	0.130	0.944

		Scenario 2.1				Scenario 2.2			
n=100	$\beta_{1}=0.3$	0.007	0.058	0.054	0.932	0.008	0.058	0.054	0.927
	$\beta_{2}=1.0$	0.026	0.210	0.199	0.937	0.028	0.209	0.197	0.934
	γ=−0.5	−0.003	0.188	0.181	0.938	−0.004	0.189	0.181	0.939
n=200	$\beta_{1}=0.3$	0.002	0.041	0.039	0.934	0.003	0.040	0.038	0.939
	$\beta_{2}=1.0$	0.011	0.147	0.141	0.938	0.013	0.144	0.139	0.939
	γ=−0.5	0.018	0.132	0.127	0.936	0.018	0.135	0.127	0.930

*Note:* Number of replications 10 000.

Abbreviations: CP, coverage probability; ESE, empirical standard deviation; sASE, average standard error based on sandwich variance estimator.

## 4 The Zurich Primary HIV Infection Study (ZPHI)

The ZPHI is an observational study at the University Hospital Zurich, Division of Infectious Diseases and Hospital Epidemiology, aiming to describe the epidemiology of primary HIV infection. More detailed information on this study can be found in Gianella et al. [23] and Von Wyl et al. [24].

Most participants in ZPHI received standard first‐line combination antiretroviral therapy (ART) shortly after the infection was confirmed, following the treatment recommendations in place at the time [25]. After maintaining viral suppression for at least 1 year, participants had the option to discontinue therapy. Treatment interruptions could also occur for several other reasons, including non‐compliance, treatment failure, toxicity, and physician discretion. Participants were closely monitored during treatment interruption, and combination ART was re‐initiated once their CD4 counts dropped below the threshold level specified by treatment guidelines of that period.

**FIGURE 3:**
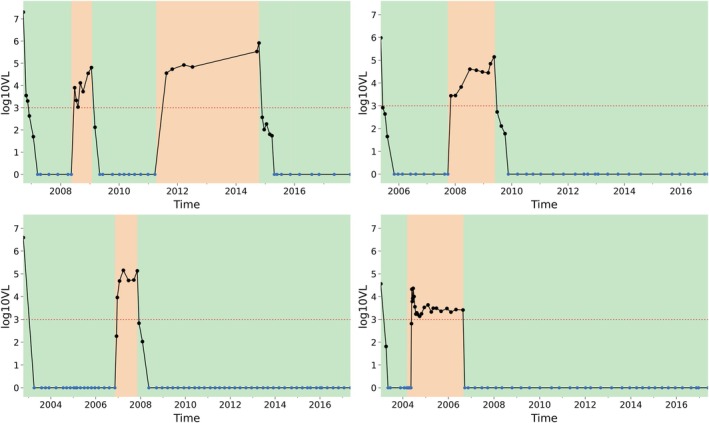
Viral load trajectories (on $\log_{10}$ scale) of four participants randomly selected from ZPHI. The green regions correspond to periods when participants were on ART while the orange regions correspond to periods when participants were off ART. Blue dots on the bottom represent the left‐censored viral load measurements by the lower quantification limit 40 copies/mL. The red dashed line is at 1000 copies/mL, corresponding to the threshold for viral rebound used in the data example.

We analyzed this dataset to investigate factors affecting viral rebound after treatment interruption. We focused on participants presenting with acute or recent HIV‐1 infection between November 2002 and July 2008, and extracted records of those who underwent treatment interruptions, defined as treatment gaps longer than 14 days during which no ART medications were taken. Gaps shorter than 14 days were excluded, as these may reflect brief interruptions due to medication switches rather than true treatment discontinuation. The final dataset includes viral load trajectories from 82 participants (9 females, 73 males). Figure 3 shows the viral trajectories (on a $\log_{10}$ scale) of 4 randomly selected study participants. A common pattern observed across participants is a steady decline in viral loads following ART initiation, followed by a rapid rebound after ART interruption.

For this application, episodes are defined by treatment phases, with an on‐ART phase followed by an off‐ART phase. Accordingly, $T_{1}$ denotes the time from ART initiation or re‐initiation to viral suppression during the on‐ART phase, and $T_{2}$ denotes the time from ART interruption to viral rebound during the subsequent off‐ART phase. We define viral suppression (Event#1) as the first time HIV RNA drops below the lower quantification limit of 40 copies/ml following ART initiation, and viral rebound (Event#2) as the first time HIV RNA exceeds 1000 copies/ml following ART interruption. The time origins (time 0) are therefore defined by treatment phase: the ART initiation/re‐initiation time for $T_{1}$ and the ART interruption time for $T_{2}$. For each endpoint, we use the observed visit/assessment times immediately before and after the threshold crossing to construct the bracketing interval. This interpretation relies on the practical data condition that the targeted first threshold crossing is localized by the observed bracket. In practice, this condition is more plausible when (i) the trajectory is approximately monotone with respect to the relevant threshold during the phase of interest (e.g., viral load declining steadily toward suppression during on‐ART), and/or (ii) monitoring is sufficiently frequent relative to the duration of any transient excursions, so that clinically meaningful threshold crossings are captured between visits. We acknowledge that intermittent monitoring could miss transient excursions or fluctuations that cross the threshold and resolve between visits.

In the first‐episode analysis (N=82 episodes), the mean bracketing‐interval length was 2.03 months (median 1.52, IQR: 1.01–2.51) for $T_{1}$ and 1.67 months (median 1.07, IQR: 0.93–2.30) for $T_{2}$. In the full‐history analysis ($N_{\text{episodes}}=159$, 82 participants), the mean bracketing‐interval length was 2.01 months (median 1.70, IQR: 1.05–2.80) for $T_{1}$ and 1.82 months (median 1.08, IQR: 0.93–2.45) for $T_{2}$. In the first‐episode analysis, all 82 episodes (100%) had interval‐censored $T_{1}$, and 81 of 82 episodes (98.8%) had interval‐censored $T_{2}$, with the remaining 1 episode (1.2%) right‐censored. In the full‐history analysis, all 159 episodes (100%) had interval‐censored $T_{1}$, while 86 episodes (54.1%) had interval‐censored $T_{2}$ and 73 episodes (45.9%) had right‐censored $T_{2}$.

In Table 3, we represent each episode by “S” if suppression information $\left(L_{1\mathrm{ij}},R_{1\mathrm{ij}}\right]$ is observed and by an additional “R” only if rebound information $\left(L_{2\mathrm{ij}},R_{2\mathrm{ij}}\right]$ is observed (i.e., $O_{2\mathrm{ij}}=1$). Thus, repeated letters (e.g., “S–S”) do not mean sustained suppression over two consecutive generic time periods; rather, they indicate consecutive episodes for which suppression is observed but rebound is unavailable in the former episode. This is a rare case in our data, but it is accommodated naturally by the clustered‐data formulation because the likelihood is defined at the episode level and allows episodes that contribute only $T_{1}$ information. Table 3 lists all five types of observed patterns of viral suppression and viral rebound in the dataset, with the majority of participants (85%) exhibiting the *suppression‐rebound‐suppression* pattern (S‐R‐S).

**TABLE 3:** Distribution of patterns of viral suppression‐rebound (S‐R) history in the ZPHI (top panel) and model fitting results from the ZPHI (bottom panel).

**Distribution of patterns of viral suppression‐rebound history (*n*)**				
S‐R	8			
S‐R‐S	70			
S‐R‐S‐R	1			
S‐R‐S‐R‐S	2			
S‐S‐R‐S‐R^a^	1			

Model fitting results	$\hat{\theta }$	$\exp\left(\hat{\theta }\right)$	Est.SE^b^	*p*
*First Episode Analysis*				
$\beta_{1}$	0.26	1.29	0.060	<0.001
$\beta_{2}$	−0.56	0.58	0.287	0.057
γ	−0.08	0.93	0.245	0.758
*Full History Analysis*				
$\beta_{1}$	0.23	1.26	0.061	<0.001
$\beta_{2}$	−0.57	0.57	0.297	0.055
γ	−0.06	0.94	0.192	0.750
*Midpoint Imputation (a)* ^c^				
$\beta_{1}$	0.26	1.30	0.096	0.007
$\beta_{2}$	−0.57	0.56	0.359	0.109

*Note:* For the proposed first‐episode and full‐history analyses, the standard error estimates (Est.SE) were computed from the observed log‐likelihood; for midpoint imputation approach (a), the reported standard errors are from the fitted interval‐censored Cox PH model.

^a^ Patterns are defined across consecutive episode‐level on‐ART/off‐ART cycles. Within episode j for individual i, $T_{1\mathrm{ij}}$ is measured from the start of the on‐ART phase and $T_{2\mathrm{ij}}$ is measured from the start of the subsequent off‐ART phase. We record S if suppression is observed (i.e., $\left(L_{1\mathrm{ij}},R_{1\mathrm{ij}}\right]$ for $T_{1\mathrm{ij}}$) and record R if rebound is observed (i.e., $\left(L_{2\mathrm{ij}},R_{2\mathrm{ij}}\right]$ for $T_{2\mathrm{ij}}$). Some episodes are partially observed, with $O_{2\mathrm{ij}}=0$, when a bracketing interval for rebound is not available. Thus, S‐S‐R‐S‐R corresponds to three episodes rather than two consecutive generic suppressed time periods: the first episode contributes only S because the off‐ART start date is missing for that episode, so rebound timing cannot be defined relative to its time origin, and episodes 2–3 each contribute S‐R.

^b^ Est.SE is the standard error of $\hat{\theta }$ (log‐hazard ratio scale).

^c^ Midpoint imputation approach (a): $T_{1}$ is imputed by the midpoint of $\left(L_{1},R_{1}\right]$, and a semiparametric interval‐censored Cox PH model for $T_{2}$ is fit via icenReg. Reported for the first‐episode analysis.

To investigate the effect of time to viral suppression on the hazard of subsequent viral rebound, we considered the following model:

$$\lambda_{2i}\left(t\right)=\lambda_{2}^{\left(0\right)}\left(t\right)\exp\left(\beta_{1}T_{1i}+\beta_{2}{\text{Female}}_{i}\right)$$

The binary covariate Female was included in addition to the time to viral suppression $T_{1}$. We postulated the model for time to viral suppression as

$$\lambda_{1i}\left(t\right)=\lambda_{1}^{\left(0\right)}\left(t\right)\exp\left(\gamma {\text{Female}}_{i}\right)$$

We first conducted a regression analysis using only the first “S‐R” episode from each patient. The independent analysis was appropriate, as each individual contributed only a single episode. We also performed an analysis using the full histories of all participants, accounting for the dependence arising from multiple episodes contributed by the same study participant. To assess the proportional hazards (PH) assumption, we generated graphical diagnostics using imputed $T_{1}$ and $T_{2}$ by plotting the estimated time‐dependent coefficients for each covariate and reporting the corresponding *p*‐values for time‐varying effects (Supporting Informations, Section D.2). The estimated effects were generally stable over follow‐up, and the tests did not indicate statistically significant time variation. Overall, these diagnostics were consistent with the PH assumption for both the time‐to‐rebound and time‐to‐suppression models.

Table 3 summarizes the parameter estimates, standard errors, and *p*‐values from the proposed *First Episode* analysis, the proposed *Full History* analysis, and, for comparison, midpoint imputation approach (a) applied to the first‐episode data. In midpoint imputation approach (a), $T_{1}$ is imputed by the midpoint of $\left(L_{1},R_{1}\right]$ and the regression for $T_{2}$ is fit using an interval‐censored Cox PH model. The midpoint‐imputation analysis produced point estimates very similar to those from the proposed first‐episode analysis. Since nearly all participants (95%) had only one viral rebound episode, the two proposed analyses (*First Episode* and *Full History*) gave very similar estimates and led to the same substantive conclusions. Overall, a longer time to viral suppression during ART was significantly associated with an increased hazard of viral rebound following ART interruption. Specifically, a one‐month increase in the time to viral suppression was associated with a 29% increase in the hazard of viral rebound in the *First Episode* analysis and a 26% increase in the *Full History* analysis. Additionally, females had approximately 42% lower hazard of viral rebound than males, conditional on the same time to viral suppression, in both proposed analyses.

## 5 Discussion

We proposed a nonparametric maximum likelihood inference method combined with a computationally stable EM algorithm for fitting proportional hazards model when both the observations on the outcome and a covariate are interval‐censored. The proposed method can be applied to both independent and clustered data settings. Our approach demonstrated satisfactory performance in simulation studies under various monitoring frequency settings. In comparison, approaches that rely on midpoint imputation for $T_{1}$ and $T_{2}$ or only for $T_{1}$ in Cox regression models can lead to suboptimal performance.

The motivating example examined the impact of the time from initiating ART treatment to achieving viral suppression ($T_{1}$) on the time from discontinuing ART to experiencing viral rebound ($T_{2}$). In standard regression settings where the covariates are fully observed, one typically postulates a model for the conditional distribution of the outcome given covariates. In the current setting where the covariate $T_{1}$ is interval‐censored, we additionally postulate a model for $T_{1}$ to jointly model the distribution of $T_{1}$ and $T_{2}$. Here, the interval‐censored $T_{1}$ was a potential predictor, occurring prior to $T_{2}$, and the two variables were defined with different time origins. Although the motivating example arises from HIV cure research, our approach is broadly applicable to settings where both the outcome and a covariate are interval‐censored observations resulting from intermittent observation schedules.

The proposed approach is applicable in settings where the key data condition holds that the observed bracketing intervals (L,R] correctly localize the event time of interest. For intermittently measured outcomes, multiple practical conditions can make this reasonable, including trajectories that are approximately monotone with respect to the relevant threshold and/or monitoring that is sufficiently frequent relative to the duration of transient excursions. In settings with much sparser monitoring or where threshold excursions are highly transient, alternative operational definitions of the endpoint, such as confirmation rules based on consecutive qualifying measurements, may be considered as sensitivity analyses when supported by the data. Our analysis framework continues to apply in settings where the endpoint is redefined in this way, because the resulting event times remain interval‐censored between assessment times, provided that the revised bracketing intervals plausibly capture the targeted event time.

The likelihood function and the fitting algorithm can be modified to allow for the assessment of effect modification. Additionally, the proposed method can be extended to accommodate multiple interval‐censored baseline covariates, allowing for the analysis of more complex data structures where several partially observed covariates influence the outcome. The proposed approach can also be adapted to handling stratification, that is, a stratum‐specific baseline hazard function can be assumed for either event process, provided that a large enough sample size is available for each stratum to ensure the hazard functions can be estimated with reasonable accuracy.

## 6 Data and Software Availability

To facilitate the application of our method, we provide an open‐source Python package along with an example input dataset and documentation at the GitHub repository (https://github.com/dli‐stats/interval_censored_covar_paper_repro). The implementation leverages JAX for efficient numerical computation and supports both independent and clustered data analyses. Users supply interval‐censored brackets $\left(L_{1},R_{1}\right]$, $\left(L_{2},R_{2}\right]$, covariate matrices, and (for clustered data) cluster identifiers; the package returns point estimates, standard errors, and confidence intervals for all regression parameters. To illustrate computational cost, the following wall‐clock times were recorded on a standard laptop (Apple M4 Pro, 48 GB memory, single core) for N=200 independent observations with a single binary covariate:

- Low‐frequency monitoring (30 visits/event; $m_{1}\approx 45$, $m_{2}\approx 55$): ∼0.06 s per fit; ∼1 min for 1000 replicates.
- High‐frequency monitoring (60 visits/event; $m_{1}\approx 90$, $m_{2}\approx 110$): ∼0.13 s per fit; ∼2 min for 1000 replicates.
- ZPHI analysis (N=82 first‐episode; $N_{\text{episodes}}=159$ full‐history): <1 s including compilation.

Computation scales roughly linearly in N and in $m_{1}m_{2}$. Runtimes increase in the clustered setting due to additional episodes and sandwich‐variance computation.

## Funding

This work was supported by National Institute of Allergy and Infectious Diseases, R01 AI170254.

## Conflicts of Interest

The authors declare no conflicts of interest.

## Supporting information


**Data S1:** Supporting Information.

## Data Availability

The data that support the findings of this study are available from University of Zurich. Restrictions apply to the availability of these data, which were used under license for this study. Data are available from the author(s) with the permission of University of Zurich.
