# Supplementary material for: Proportional Hazards Regression for Interval‐Censored Outcomes With an Interval‐Censored Covariate
Source: Stat Med. 2026 May 4;45:e70573. doi: 10.1002/sim.70573 (PMC13136924; doi:10.1002/sim.70573)
Supplement: Supplementary file 1 — Data S1: Supporting Information. [file SIM-45-0-s001.pdf]

# APPENDIX A: POISSON AUGMENTATION AND EM ALGORITHM FOR THE INDEPENDENT DATA SETTING

## A.1 Augmented data likelihood

Following Zeng et al.[1], we introduce latent variables $W_{ik}$, $i = 1, \dots, N, k = 1, \dots, m_2$, which, conditional on $(T_{1i}, \boldsymbol{Z}_{2i})$, are independent Poisson random variables with means $\lambda_{2k} \exp(\beta_1 T_{1i} + \boldsymbol{\beta}_2^\top \boldsymbol{Z}_{2i})$. In addition, let

$$A_i = \sum_{t_{2k} \leq L_{2i}} W_{ik}, \text{ and } B_i = I(R_{2i} < \infty) \sum_{L_{2i} < t_{2k} \leq R_{2i}} W_{ik}$$

An equivalent expression of the observed data likelihood can be constructed using the $W_{ik}$'s, where the event $T_{2i} \in (L_{2i}, R_{2i}]$ is equivalent to $\{A_i = 0\} \cap \{B_i > 0\}$ for $R_{2i} < \infty$ and $\{A_i = 0\}$ for $R_{2i} = \infty$. Since the distribution of $T_1$ is discretized, we use $I_{i\ell}$ to denote the indicator $I(T_{1i} = t_{1\ell} | \boldsymbol{z}_{1i})$. Define the augmented data collection for individual $i$ by

$$\mathcal{A}_i = \{I_{i\ell}, \boldsymbol{z}_{1i}, \boldsymbol{z}_{2i}, W_{ik} \,:\, \ell = 1, .., m_1, k = 1, ..., m_2\}.$$

Denote $R_{2i}^* = L_{2i} I(R_{2i} = \infty) + R_{2i} I(R_{2i} < \infty)$. The likelihood function of the augmented data $\mathcal{A} = \cup_{i=1}^n \mathcal{A}_i$ is then given by:

$$\mathcal{L}_{aug}(\boldsymbol{\theta}; \mathcal{A}) = \prod_{i=1}^N \sum_{L_{1i} < t_{1\ell} \leq R_{1i}} I_{i\ell} e^{-\exp(\boldsymbol{\gamma}^\top \boldsymbol{z}_{1i}) \sum_{l' \leq l-1} \lambda_{1l'}} \left(1 - e^{-\exp(\boldsymbol{\gamma}^\top \boldsymbol{z}_{1i}) \lambda_{1l}}\right) \times \left\{ \prod_{t_{2k} \leq R_{2i}^*} \frac{\exp[-\lambda_{2k} \exp(\beta_1 t_{1\ell} + \boldsymbol{\beta}_2^\top \boldsymbol{z}_{2i})][\lambda_{2k} \exp(\beta_1 t_{1\ell} + \boldsymbol{\beta}_2^\top \boldsymbol{z}_{2i})]^{W_{ik}}}{W_{ik}!} \right\}$$

and the log-likelihood of the augmented data is then, up to a constant,

$$\log \mathcal{L}_{aug}(\boldsymbol{\theta}; \mathcal{A}) = \sum_{i=1}^N \sum_{L_{1i} < t_{1\ell} \leq R_{1i}} I_{i\ell} \Big\{ -\exp(\boldsymbol{\gamma}^\top \boldsymbol{z}_{1i}) \sum_{l' \leq l-1} \lambda_{1l'} + \log\left(1 - e^{-\exp(\boldsymbol{\gamma}^\top \boldsymbol{z}_{1i}) \lambda_{1l}}\right) + \sum_{t_{2k} \leq R_{2i}^*} W_{ik} \left(\log \lambda_{2k} + \beta_1 t_{1\ell} + \boldsymbol{\beta}_2^\top \boldsymbol{z}_{2i}\right) - \lambda_{2k} \exp(\beta_1 t_{1\ell} + \boldsymbol{\beta}_2^\top \boldsymbol{z}_{2i}) \Big\}.$$

Let $\boldsymbol{\theta}_r$ be the current estimate of $\boldsymbol{\theta}$. The expectation step (E-step) computes the conditional expectation of the augmented data log-likelihood given the observed data and current estimate:

$$Q(\boldsymbol{\theta}|\boldsymbol{\theta}_r) = \sum_{i=1}^N \sum_{L_{1i} < t_{1\ell} \leq R_{1i}} \Big\{ \mathbb{E}_{\boldsymbol{\theta}_r}[I_{i\ell} | \mathcal{D}_i] \Big[ -\exp(\boldsymbol{\gamma}^\top \boldsymbol{z}_{1i}) \sum_{l' \leq l-1} \lambda_{1l'} + \log\left(1 - e^{-\exp(\boldsymbol{\gamma}^\top \boldsymbol{z}_{1i}) \lambda_{1l}}\right) \Big] + \sum_{t_{2k} \leq R_{2i}^*} \Big[ \mathbb{E}_{\boldsymbol{\theta}_r}[W_{ik} I_{i\ell} | \mathcal{D}_i] \big( \log \lambda_{2k} + \beta_1 t_{1\ell} + \boldsymbol{\beta}_2^\top \boldsymbol{z}_{2i} \big) - \mathbb{E}_{\boldsymbol{\theta}_r}[I_{i\ell} | \mathcal{D}_i] \lambda_{2k} \exp(\beta_1 t_{1\ell} + \boldsymbol{\beta}_2^\top \boldsymbol{z}_{2i}) \Big] \Big\} \quad (1)$$

where

$$\hat{p}_{il} \triangleq \mathbb{E}_{\boldsymbol{\theta}_r}[I_{i\ell} | \mathcal{D}_i; \boldsymbol{\theta}] = \frac{e^{-\exp(\boldsymbol{\gamma}_r^\top \boldsymbol{z}_{1i}) \sum_{l' \leq l-1} \lambda_{1l',r}} \left(1 - e^{-\exp(\boldsymbol{\gamma}_r^\top \boldsymbol{z}_{1i}) \lambda_{1l,r}}\right) \Big[ S_l(L_{2i}) - I(R_{2i} < \infty) S_l(R_{2i}) \Big]}{\sum_{L_{1i} < t_{1g} \leq R_{1i}} e^{-\exp(\boldsymbol{\gamma}_r^\top \boldsymbol{z}_{1i}) \sum_{l' \leq g-1} \lambda_{1l',r}} \left(1 - e^{-\exp(\boldsymbol{\gamma}_r^\top \boldsymbol{z}_{1i}) \lambda_{1g,r}}\right) \Big[ S_g(L_{2i}) - I(R_{2i} < \infty) S_g(R_{2i}) \Big]}$$

for $t_{1\ell} \in (L_{1i}, R_{1i}]$, and $\hat{p}_{il} = 0$ otherwise. Here $S_l(\eta) \triangleq e^{-\exp(\beta_{1,r} t_{1\ell} + \boldsymbol{\beta}_{2,r}^\top \boldsymbol{z}_{2i}) \sum_{t_{2k} \leq \eta} \lambda_{2k,r}}$, and $S_g(\eta) \triangleq e^{-\exp(\beta_{1,r} t_{1g} + \boldsymbol{\beta}_{2,r}^\top \boldsymbol{z}_{2i}) \sum_{t_{2k} \leq \eta} \lambda_{2k,r}}$. Additionally, we have

$$\widehat{W}_{ikl} \triangleq \mathbb{E}_{\theta_r}[W_{ik} I_{i\ell} | \mathcal{D}_i] = \widehat{p}_{il} \times \frac{\lambda_{2k,r} \exp(\beta_{1,r} t_{1\ell} + \boldsymbol{\beta}_{2,r}^\top \boldsymbol{z}_{2i})}{1 - \exp\left(-\sum_{L_{2i} < t_{2k'} \leq R_{2i}} \lambda_{2k',r} \exp(\beta_{1,r} t_{1\ell} + \boldsymbol{\beta}_{2,r}^\top \boldsymbol{z}_{2i})\right)}$$

for $t_{2k} \in (L_{2i}, R_{2i}]$ and $\widehat{W}_{ikl} = 0$ otherwise.

## A.2 M-step score equations

To update the parameter estimates, the M-step maximizes $Q(\boldsymbol{\theta}|\boldsymbol{\theta}_r)$. Differentiating with respect to $\lambda_{1\ell}$ and equating to 0:

$$\sum_{i=1}^{N} \widehat{p}_{il} I(L_{1i} < t_{1l} \leq R_{1i}) \frac{e^{\boldsymbol{\gamma}^\top \boldsymbol{z}_{1i}} e^{-\exp(\boldsymbol{\gamma}^\top \boldsymbol{z}_{1i})\lambda_{1l}}}{1 - e^{-\exp(\boldsymbol{\gamma}^\top \boldsymbol{z}_{1i})\lambda_{1l}}} - \sum_{i=1}^{N} \sum_{l'>l} \widehat{p}_{il'} I(L_{1i} < t_{1l'} \leq R_{1i}) e^{\boldsymbol{\gamma}^\top \boldsymbol{z}_{1i}} = 0$$

Similarly, differentiating (1) with respect to $\boldsymbol{\gamma}$:

$$\sum_{i=1}^{N} \sum_{L_{1i} < t_{1l} \leq R_{1i}} \widehat{p}_{il} \left\{ \boldsymbol{z}_{1i} e^{\boldsymbol{\gamma}^\top \boldsymbol{z}_{1i}} \sum_{l' \leq l-1} \lambda_{1l'} - \frac{e^{-\exp(\boldsymbol{\gamma}^\top \boldsymbol{z}_{1i})\lambda_{1l}} e^{\boldsymbol{\gamma}^\top \boldsymbol{z}_{1i}} \lambda_{1l}}{1 - e^{-\exp(\boldsymbol{\gamma}^\top \boldsymbol{z}_{1i})\lambda_{1l}}} \right\} = \mathbf{0}$$

The above two sets of score equations can be solved using the Newton–Raphson method. Denote the solutions as $\lambda_{1l,(r+1)}$ and $\boldsymbol{\gamma}_{r+1}$. Differentiating (1) with respect to $\lambda_{2k}$ and equating to 0:

$$\widehat{\lambda}_{2k}(\beta_1, \boldsymbol{\beta}_2) = \frac{\sum_{i=1}^{N} \sum_{L_{1i} < t_{1\ell} \leq R_{1i}} I(t_{2k} \leq R_{2i}^*) \widehat{W}_{ik\ell}}{\sum_{i=1}^{N} \sum_{L_{1i} < t_{1\ell} \leq R_{1i}} \left\{ I(t_{2k} \leq R_{2i}^*) \widehat{p}_{i\ell} \exp(\beta_1 t_{1\ell} + \boldsymbol{\beta}_2^\top \boldsymbol{z}_{2i}) \right\}} \tag{2}$$

Incorporating (2) into (1) and differentiating with respect to $(\beta_1, \boldsymbol{\beta}_2^\top)^\top$:

$$\sum_{i=1}^{N} \sum_{L_{1i} < t_{1\ell} \leq R_{1i}} \sum_{t_{2k} \leq R_{2i}^*} \left[ \widehat{W}_{ikl} - \widehat{p}_{il} \exp\left( \log \widehat{\lambda}_{2k}(\beta_1, \boldsymbol{\beta}_2) + \beta_1 t_{1l} + \boldsymbol{\beta}_2^\top \boldsymbol{z}_{2i} \right) \right] \times$$

$$\left\{ \begin{pmatrix} t_{1l} \\ \boldsymbol{z}_{2i} \end{pmatrix} - \frac{\sum_{i'=1}^{N} \sum_{L_{1i'} < t_{1l'} \leq R_{1i'}} \sum_{t_{2k'} \leq R_{2i'}^*} \widehat{p}_{i'l'} \exp(\beta_1 t_{1l'} + \boldsymbol{\beta}_2^\top \boldsymbol{z}_{2i'}) \begin{pmatrix} t_{1l'} \\ \boldsymbol{z}_{2i'} \end{pmatrix}}{\sum_{i'=1}^{N} \sum_{L_{1i'} < t_{1l'} \leq R_{1i'}} \sum_{t_{2k'} \leq R_{2i'}^*} \widehat{p}_{i'l'} \exp(\beta_1 t_{1l'} + \boldsymbol{\beta}_2^\top \boldsymbol{z}_{2i'})} \right\} = \mathbf{0}$$

The score equations are solved via Newton–Raphson. Denote the solutions as $\beta_{1,r+1}$ and $\boldsymbol{\beta}_{2,r+1}$; substituting back into (2) gives $\lambda_{21,(r+1)}, \dots, \lambda_{2m_2,(r+1)}$. The algorithm iterates between E-step and M-step until convergence.

## A.3 Derivations of E-step conditional expectations

Denote the current estimates of $\beta_1$, $\boldsymbol{\beta}_2$, $\boldsymbol{\gamma}$, $\lambda_{1\ell}$ and $\lambda_{2k}$ as $\beta_{1r}$, $\boldsymbol{\beta}_{2r}$, $\boldsymbol{\gamma}_r$, $\lambda_{1l,r}$ and $\lambda_{2k,r}$, respectively.

1. Update $\widehat{p}_{il}$:

   (a) For $t_{1l} \in (L_{1i}, R_{1i}]$, $R_{2i} < \infty$:

$$\begin{aligned}
\widehat{p}_{il} &= E[I(T_{1i} = t_{1l}) \mid \mathcal{D}_i, \beta_{1r}, \boldsymbol{\beta}_{2r}, \boldsymbol{\gamma}_r] \\
&= \frac{P(T_{1i} = t_{1\ell}) P(L_{2i} < T_{2i} \leq R_{2i} \mid T_{1i} = t_{1\ell})}{\sum_{L_{1i} < t_{1g} \leq R_{1i}} P(T_{1i} = t_{1g}) P(L_{2i} < T_{2i} \leq R_{2i} \mid T_{1i} = t_{1g})} \\
&= \frac{\lambda_{1l,r} e^{-\exp(\boldsymbol{\gamma}_r^\top \boldsymbol{z}_{1i}) \sum_{\ell' \leq \ell - 1} \lambda_{1\ell',r}} \left[ e^{-\exp(\beta_{1r} t_{1\ell} + \boldsymbol{\beta}_{2r}^\top \boldsymbol{z}_{2i}) \sum_{t_{2k} \leq L_{2i}} \lambda_{2k,r}} - e^{-\exp(\beta_{1r} t_{1\ell} + \boldsymbol{\beta}_{2r}^\top \boldsymbol{z}_{2i}) \sum_{t_{2k} \leq R_{2i}} \lambda_{2k,r}} \right]}{\sum_{L_{1i} < t_{1g} \leq R_{1i}} \lambda_{1g,r} e^{-\exp(\boldsymbol{\gamma}_r^\top \boldsymbol{z}_{1i}) \sum_{g' \leq g-1} \lambda_{1g',r}} \left[ e^{-\exp(\beta_{1r} t_{1g} + \boldsymbol{\beta}_{2r}^\top \boldsymbol{z}_{2i}) \sum_{t_{2k} \leq L_{2i}} \lambda_{2k,r}} - e^{-\exp(\beta_{1r} t_{1g} + \boldsymbol{\beta}_{2r}^\top \boldsymbol{z}_{2i}) \sum_{t_{2k} \leq R_{2i}} \lambda_{2k,r}} \right]}
\end{aligned}$$

(b) For $t_{1\ell} \in (L_{1i}, R_{1i}]$, $R_{2i} = \infty$:

$$\begin{aligned}
\widehat{p}_{i\ell} &= E[I(T_{1i} = t_{1\ell}) \mid \mathcal{D}_i, \beta_{1r}, \boldsymbol{\beta}_{2r}, \boldsymbol{\gamma}_r] \\
&= \frac{P(T_{1i} = t_{1\ell})P(T_{2i} > L_{2i} \mid T_{1i} = t_{1\ell})}{\sum_{L_{1i} < t_{1g} \le R_{1i}} P(T_{1i} = t_{1g})P(T_{2i} > L_{2i} \mid T_{1i} = t_{1g})} \\
&= \frac{\lambda_{1\ell,r} e^{-\exp(\boldsymbol{\gamma}_r^\top \boldsymbol{z}_{1i}) \sum_{\ell' \le \ell-1} \lambda_{1\ell',r}} [e^{-\exp(\beta_{1r} t_{1\ell} + \boldsymbol{\beta}_{2r}^\top \boldsymbol{z}_{2i}) \sum_{t_{2k} \le L_{2i}} \lambda_{2k,r}}]}{\sum_{L_{1i} < t_{1g} \le R_{1i}} \lambda_{1g,r} e^{-\exp(\boldsymbol{\gamma}_r^\top \boldsymbol{z}_{1i}) \sum_{g' \le g-1} \lambda_{1g',r}} [e^{-\exp(\beta_{1r} t_{1g} + \boldsymbol{\beta}_{2r}^\top \boldsymbol{z}_{2i}) \sum_{t_{2k} \le L_{2i}} \lambda_{2k,r}}]}
\end{aligned}$$

(c) For $t_{1\ell} \notin (L_{1i}, R_{1i}]$: $\widehat{p}_{i\ell} = 0$;

2. Update $\widehat{W}_{ik\ell}$:
Recall that $W_{ik} | T_{1i}, \boldsymbol{Z}_{2i} \sim Poisson(\lambda_{2k} \exp(\beta_1 T_{1i} + \boldsymbol{\beta}_2^\top \boldsymbol{Z}_{2i}))$

(a) For $t_{2k} \le L_{2i}$, $\widehat{W}_{ik\ell} = 0$;

(b) For $t_{2k} \in (L_{2i}, R_{2i}]$, $R_{2i} < \infty, t_{1\ell} \in (L_{1i}, R_{1i}]$:

$$\begin{aligned}
\widehat{W}_{ik\ell} &= E[I(T_{1i} = t_{1\ell}) W_{ik} \mid \mathcal{D}_i, \beta_{1r}, \boldsymbol{\beta}_{2r}, \boldsymbol{\gamma}_r] \\
&= E\left\{ E[I(T_{1i} = t_{1\ell}) W_{ik} | T_{1i}, L_{2i} < T_{2i} \le R_{2i}, \beta_{1r}, \boldsymbol{\beta}_{2r}, \boldsymbol{\gamma}_r] \right\} \\
&= E\left\{ I(T_{1i} = t_{1\ell}) E[W_{ik} | T_{1i}, \sum_{L_{2i} < t_{2k'} \le R_{2i}} W_{ik'} > 0, \beta_{1r}, \boldsymbol{\beta}_{2r}, \boldsymbol{\gamma}_r] \right\} \\
&= \widehat{p}_{i\ell} \times E[W_{ik} | T_{1i} = t_{1\ell}, \sum_{L_{2i} < t_{2k'} \le R_{2i}} W_{ik'} > 0, \beta_{1r}, \boldsymbol{\beta}_{2r}, \boldsymbol{\gamma}_r] \\
&= \widehat{p}_{i\ell} \times \frac{\lambda_{2k,r} \exp(\beta_{1r} t_{1\ell} + \boldsymbol{\beta}_{2r}^\top \boldsymbol{z}_{2i})}{1 - \exp(-\sum_{L_{2i} < t_{2k'} \le R_{2i}} \lambda_{2k',r} \exp(\beta_{1r} t_{1\ell} + \boldsymbol{\beta}_{2r}^\top \boldsymbol{z}_{2i}))}
\end{aligned}$$

(c) For $t_{2k} \in (L_{2i}, R_{2i}], t_{1\ell} \notin (L_{1i}, R_{1i}]$, $\widehat{W}_{ik\ell} = 0$.

## APPENDIX B: COMPOSITE EM ALGORITHM FOR THE CLUSTERED DATA SETTING

Introduce latent variables $W_{ijk}$, $i = 1, \dots, N, j = 1, \dots, n_i, k = 1, \dots, m_2$, which, conditional on $(T_{1ij}, \boldsymbol{Z}_{2ij})$, are independent Poisson random variables with means $\lambda_{2k} \exp(\beta_1 T_{1ij} + \boldsymbol{\beta}_2^\top \boldsymbol{Z}_{2ij})$. In addition, let $I_{ij\ell}$ denote the indicator $I(T_{1ij} = t_{1\ell} | \boldsymbol{z}_{1i})$. Define the augmented data collection for episode $j$ of individual $i$ by

$$\mathcal{A}_{ij} = \{I_{ij\ell}, \boldsymbol{z}_{2ij}, \boldsymbol{z}_{1ij}, O_{2ij} W_{ijk}, O_{2ij} \,:\, \ell = 1, .., m_1, k = 1, ..., m_2\}$$

The composite log-likelihood for the augmented data $\mathcal{A}_C \triangleq \cup_{i=1}^n \cup_{j=1}^{n_i} \mathcal{A}_{ij}$ is, up to a constant,

$$\begin{aligned}
\log \mathcal{L}_C(\boldsymbol{\theta}; \mathcal{A}_C) = \sum_{i=1}^N \sum_{j=1}^{n_i} \sum_{L_{1ij} < t_{1\ell} \le R_{1ij}} I_{ij\ell} \Bigg\{ &- \exp(\boldsymbol{\gamma}^\top \boldsymbol{z}_{1ij}) \sum_{l' \le l-1} \lambda_{1l'} + \log\left(1 - e^{-\exp(\boldsymbol{\gamma}^\top \boldsymbol{z}_{1ij}) \lambda_{1l}}\right) \\
&+ O_{2ij} \left[ \sum_{t_{2k} \le R_{2ij}^*} W_{ijk} \left(\log \lambda_{2k} + \beta_1 t_{1\ell} + \boldsymbol{\beta}_2^\top \boldsymbol{z}_{2ij}\right) - \lambda_{2k} \exp(\beta_1 t_{1\ell} + \boldsymbol{\beta}_2^\top \boldsymbol{z}_{2ij}) \right] \Bigg\}
\end{aligned}$$

Let $\boldsymbol{\theta}_r$ be the current estimate of $\boldsymbol{\theta}$. The expectation step computes the conditional expectation of the augmented data log-likelihood given the observed data and current estimates. That is,

$$Q_C(\boldsymbol{\theta} \mid \boldsymbol{\theta}_r) = \sum_{i=1}^{N}\sum_{j=1}^{n_i}\sum_{L_{1ij}<t_{1\ell}\le R_{1ij}} \Bigg\{ \mathbb{E}_{\theta_r}[I_{ij\ell}|\mathcal{D}_{ij}]\left(-\exp(\boldsymbol{\gamma}^\top \boldsymbol{z}_{1ij})\sum_{l'\le l-1}\lambda_{1l'} + \log\left(1-e^{-\exp(\boldsymbol{\gamma}^\top \boldsymbol{z}_{1ij})\lambda_{1l}}\right)\right)$$
$$+ O_{2ij}\left[\sum_{t_{2k}\le R^*_{2ij}}\left(\mathbb{E}_{\boldsymbol{\theta}_r}[W_{ijk}I_{ij\ell}|\mathcal{D}_{ij}]\left(\log\lambda_{2k}+\beta_{1r}t_{1\ell}+\boldsymbol{\beta}_{2r}^\top \boldsymbol{z}_{2ij}\right) - \mathbb{E}_{\boldsymbol{\theta}_r}[I_{ij\ell}|\mathcal{D}_{ij}]\lambda_{2k}\exp(\beta_{1r}t_{1\ell}+\boldsymbol{\beta}_{2r}^\top \boldsymbol{z}_{2ij})\right)\right]\Bigg\} \quad (3)$$

We will use shorthand $\widehat{p}_{ij\ell}$ for $\mathbb{E}_{\boldsymbol{\theta}_r}[I_{ij\ell}|\mathcal{D}_{ij}]$ and $\widehat{W}_{ijk\ell}$ for $\mathbb{E}_{\boldsymbol{\theta}_r}[W_{ijk}I_{ij\ell}|\mathcal{D}_{ij}]$ henceforth for simplicity of notation.

Denote the current estimates of $\beta_1$, $\boldsymbol{\beta}_2$, $\boldsymbol{\gamma}$, $\lambda_{1\ell}$ and $\lambda_{2k}$ as $\beta_{1r}$, $\boldsymbol{\beta}_{2r}$, $\boldsymbol{\gamma}_r$, $\lambda_{1l,r}$ and $\lambda_{2k,r}$, respectively.

1. Update $\widehat{p}_{ij\ell}$:

   (a) For $t_{1\ell} \in (L_{1ij}, R_{1ij}]$, $R_{2ij} < \infty$:

$$\widehat{p}_{ij\ell} = E[I(T_{1ij} = t_{1\ell}) \mid \mathcal{D}_{ij}, \beta_{1r}, \boldsymbol{\beta}_{2r}, \boldsymbol{\gamma}_r]$$
$$= \frac{P(T_{1ij}=t_{1\ell})\left[P(L_{2ij}<T_{2ij}\le R_{2ij}\mid T_{1ij}=t_{1\ell})\right]^{O_{2ij}}}{\sum_{L_{1ij}<t_{1g}\le R_{1ij}} P(T_{1ij}=t_{1g})\left[P(L_{2ij}<T_{2ij}\le R_{2ij}\mid T_{1ij}=t_{1g})\right]^{O_{2ij}}}$$

   with the numerator evaluating to

$$\lambda_{1\ell,r}e^{-e^{(\gamma_r^\top z_{1ij})}\sum_{\ell'\le\ell-1}\lambda_{1\ell',r}}[e^{-\exp(\beta_{1r}t_{1\ell}+\beta_{2r}^\top z_{2ij})\sum_{t_{2k}\le L_{2ij}}\lambda_{2k,r}} - e^{-\exp(\beta_{1r}t_{1\ell}+\beta_{2r}^\top z_{2ij})\sum_{t_{2k}\le R_{2ij}}\lambda_{2k,r}}]^{O_{2ij}}$$

   and the denominator evaluating to

$$\sum_{L_{1ij}<t_{1g}\le R_{1ij}}\lambda_{1g,r}e^{-e^{(\gamma_r^\top z_{1ij})}\sum_{g'\le g-1}\lambda_{1g',r}}[e^{-\exp(\beta_{1r}t_{1g}+\beta_{2r}^\top z_{2ij})\sum_{t_{2k}\le L_{2ij}}\lambda_{2k,r}} - e^{-\exp(\beta_{1r}t_{1g}+\beta_{2r}^\top z_{2ij})\sum_{t_{2k}\le R_{2ij}}\lambda_{2k,r}}]^{O_{2ij}}$$

   (b) For $t_{1\ell} \in (L_{1ij}, R_{1ij}]$, $R_{2ij} = \infty$:

$$\widehat{p}_{ij\ell} = E[I(T_{1ij} = t_{1\ell}) \mid \mathcal{D}_{ij}, \beta_{1r}, \boldsymbol{\beta}_{2r}, \boldsymbol{\gamma}_r]$$
$$= \frac{P(T_{1ij}=t_{1\ell})\left[P(T_{2ij}>L_{2ij}\mid T_{1ij}=t_{1\ell})\right]^{O_{2ij}}}{\sum_{L_{1ij}<t_{1g}\le R_{1ij}} P(T_{1ij}=t_{1g})\left[P(T_{2ij}>L_{2ij}\mid T_{1ij}=t_{1g})\right]^{O_{2ij}}}$$
$$= \frac{\lambda_{1\ell,r}e^{-\exp(\boldsymbol{\gamma}_r^\top \boldsymbol{z}_{1ij})\sum_{\ell'\le\ell-1}\lambda_{1\ell',r}}[e^{-\exp(\beta_{1r}t_{1\ell}+\boldsymbol{\beta}_{2r}^\top \boldsymbol{z}_{2ij})\sum_{t_{2k}\le L_{2ij}}\lambda_{2k,r}}]^{O_{2ij}}}{\sum_{L_{1ij}<t_{1g}\le R_{1ij}}\lambda_{1g,r}e^{-\exp(\boldsymbol{\gamma}_r^\top \boldsymbol{z}_{1ij})\sum_{g'\le g-1}\lambda_{1g',r}}[e^{-\exp(\beta_{1r}t_{1g}+\boldsymbol{\beta}_{2r}^\top \boldsymbol{z}_{2ij})\sum_{t_{2k}\le L_{2ij}}\lambda_{2k,r}}]^{O_{2ij}}}$$

   (c) For $t_{1\ell} \notin (L_{1ij}, R_{1ij}]$: $\widehat{p}_{ij\ell} = 0$;

2. Update $\widehat{W}_{ijk\ell}$:
   Recall that $W_{ijk} \mid T_{1ij}, \boldsymbol{Z}_{2ij} \sim Poisson(\lambda_{2k}\exp(\beta_1 T_{1ij} + \boldsymbol{\beta}_2^\top \boldsymbol{Z}_{2ij}))$

   (a) For $t_{2k} \le L_{2ij}$, $\widehat{W}_{ijk\ell} = 0$;

(b) For $t_{2k} \in (L_{2ij}, R_{2ij}], R_{2ij} < \infty, t_{1\ell} \in (L_{1ij}, R_{1ij}]$:

$$\begin{aligned}
\widehat{W}_{ijk\ell} &= E[I(T_{1ij} = t_{1\ell})W_{ijk} \mid \mathcal{D}_{ij}, \beta_{1r}, \boldsymbol{\beta}_{2r}, \boldsymbol{\gamma}_r] \\
&= E\left\{ E[I(T_{1ij} = t_{1\ell})W_{ijk} | T_{1ij}, L_{2ij} < T_{2ij} \le R_{2ij}, \beta_{1r}, \boldsymbol{\beta}_{2r}, \boldsymbol{\gamma}_r] \right\} \\
&= E\left\{ I(T_{1ij} = t_{1\ell})E[W_{ijk} | T_{1ij}, \sum_{L_{2ij} < t_{2k'} \le R_{2ij}} W_{ijk'} > 0, \beta_{1r}, \boldsymbol{\beta}_{2r}, \boldsymbol{\gamma}_r] \right\} \\
&= \widehat{p}_{ij\ell} \times E[W_{ijk} \mid T_{1ij} = t_{1\ell}, \sum_{L_{2ij} < t_{2k'} \le R_{2ij}} W_{ijk'} > 0, \beta_{1r}, \boldsymbol{\beta}_{2r}, \boldsymbol{\gamma}_r] \\
&= \widehat{p}_{ij\ell} \times \frac{\lambda_{2k,r} \exp(\beta_{1r} t_{1\ell} + \boldsymbol{\beta}_{2r}^\top \boldsymbol{z}_{2ij})}{1 - \exp(-\sum_{L_{2ij} < t_{2k'} \le R_{2ij}} \lambda_{2k',r} \exp(\beta_{1r} t_{1\ell} + \boldsymbol{\beta}_{2r}^\top \boldsymbol{z}_{2ij}))}
\end{aligned}$$

(c) For $t_{2k} \in (L_{2ij}, R_{2ij}], t_{1\ell} \notin (L_{1ij}, R_{1ij}], \widehat{W}_{ijk\ell} = 0$.

In the M-step, we update the parameter estimates through maximizing $Q_C(\boldsymbol{\theta}|\boldsymbol{\theta}_r)$. First, differentiating with respect to $\lambda_{1\ell}, \ell = 1, ..., m_1$ and setting the derivative to 0 yields the following score equation:

$$\sum_{i=1}^{N} \sum_{j=1}^{n_i} \widehat{p}_{ijl} I(L_{1ij} < t_{1l} \le R_{1ij}) \frac{e^{\boldsymbol{\gamma}^\top \boldsymbol{z}_{1ij}} e^{-\exp(\boldsymbol{\gamma}^\top \boldsymbol{z}_{1ij})\lambda_{1l}}}{1 - e^{-\exp(\boldsymbol{\gamma}^\top \boldsymbol{z}_{1ij})\lambda_{1l}}} - \sum_{i=1}^{N} \sum_{j=1}^{n_i} \sum_{l' > l} \widehat{p}_{ijl'} I(L_{1ij} < t_{1l'} \le R_{1ij}) e^{\boldsymbol{\gamma}^\top \boldsymbol{z}_{1ij}} = 0$$

Similarly, differentiating (3) with respect to $\boldsymbol{\gamma}$, we obtain the following score equation:

$$\sum_{i=1}^{N} \sum_{j=1}^{n_i} \sum_{L_{1ij} < t_{1l} \le R_{1ij}} \widehat{p}_{ijl} \left\{ \boldsymbol{z}_{1ij} e^{\boldsymbol{\gamma}^\top \boldsymbol{z}_{1ij}} \sum_{l' \le l-1} \lambda_{1l'} - \frac{e^{-\exp(\boldsymbol{\gamma}^\top \boldsymbol{z}_{1ij})\lambda_{1l}} e^{\boldsymbol{\gamma}^\top \boldsymbol{z}_{1ij}} \lambda_{1l}}{1 - e^{-\exp(\boldsymbol{\gamma}^\top \boldsymbol{z}_{1ij})\lambda_{1l}}} \right\} = \boldsymbol{0}$$

The above two sets of score equations can be solved using the Newton-Raphson method. Denote the solutions as $\lambda_{1l,(r+1)}$, $l = 1, \ldots, m_1$, and $\boldsymbol{\gamma}_{r+1}$.

By differentiating (3) with respect to $\lambda_{2k}$ and equating to 0, we obtain the following closed form:

$$\widehat{\lambda}_{2k} = \frac{\sum_{i=1}^{N} \sum_{j=1}^{n_i} \sum_{L_{1ij} < t_{1\ell} \le R_{1ij}} O_{2ij} I(t_{2k} \le R_{2ij}^*) \widehat{W}_{ijk\ell}}{\sum_{i=1}^{N} \sum_{j=1}^{n_i} \sum_{L_{1ij} < t_{1\ell} \le R_{1ij}} O_{2ij} I(t_{2k} \le R_{2ij}^*) \widehat{p}_{ij\ell} \exp(\beta_1 t_{1\ell} + \boldsymbol{\beta}_2^\top \boldsymbol{z}_{2ij})} \tag{4}$$

Incorporating (4) into (3) and differentiating with respect to $(\beta_1, \boldsymbol{\beta}_2^\top)^\top$, we obtain the following score equation:

$$\sum_{i=1}^{N} \sum_{j=1}^{n_i} \sum_{L_{1ij} < t_{1\ell} \le R_{1ij}} \sum_{t_{2k} \le R_{2ij}^*} \left[ \widehat{W}_{ijkl} - \widehat{p}_{ijl} \exp\left( \log \widehat{\lambda}_{2k}(\beta_1, \boldsymbol{\beta}_2) + \beta_1 t_{1l} + \boldsymbol{\beta}_2^\top \boldsymbol{z}_{2ij} \right) \right] \times$$

$$\left\{ \begin{pmatrix} t_{1l} \\ \boldsymbol{z}_{2ij} \end{pmatrix} - \frac{\sum_{i'=1}^{N} \sum_{j'=1}^{n_{i'}} \sum_{L_{1i'j'} < l' \le R_{1i'j'}} \sum_{t_{2k'} \le R_{2i'j'}^*} \widehat{p}_{i'j'l'} \exp(\beta_1 t_{1l'} + \boldsymbol{\beta}_2^\top \boldsymbol{z}_{2i'j'}) \begin{pmatrix} t_{1l} \\ \boldsymbol{z}_{2ij} \end{pmatrix}}{\sum_{i'=1}^{N} \sum_{j'=1}^{n_{i'}} \sum_{L_{1i'j'} < l' \le R_{1i'j'}} \sum_{t_{2k'} \le R_{2i'j'}^*} \widehat{p}_{i'j'l'} \exp(\beta_1 t_{1l'} + \boldsymbol{\beta}_2^\top \boldsymbol{z}_{2i'j'})} \right\} = \boldsymbol{0}$$

The score equation can be solved using the Newton-Raphson method. Denote the solutions as $\beta_{1,r+1}$ and $\boldsymbol{\beta}_{2,r+1}$, and plug them back into (4) to obtain the updated estimates $\lambda_{21,(r+1)}, ..., \lambda_{2m_2,(r+1)}$. The algorithm iterates between the E-step and M-step until convergence.

## APPENDIX C: ADDITIONAL DETAILS FOR ASYMPTOTIC PROPERTIES

The asymptotic properties of the proposed estimator $(\hat{\boldsymbol{\xi}}, \widehat{\Lambda}_1, \widehat{\Lambda}_2)$ are established under the following conditions. Here, $\boldsymbol{\xi} = (\beta_1, \boldsymbol{\beta}_2^\top, \boldsymbol{\gamma}^\top)^\top$ denotes the vector of regression coefficients, and $\Lambda_1(\cdot)$ and $\Lambda_2(\cdot)$ denote the cumulative baseline hazard functions for $T_1$ and $T_2$, respectively. Define the following function:

$$W(t_1, t_2, \boldsymbol{\xi}, \Lambda_2) = \exp\left[-\exp(\beta_1 t_1 + \boldsymbol{\beta}_2^\top \boldsymbol{Z}_2)\Lambda_2(t_2)\right] \exp\left[-\exp(\boldsymbol{\gamma}^\top \boldsymbol{Z}_1)\Lambda_1(t_1)\right] \lambda_1(t_1) \exp(\boldsymbol{\gamma}^\top \boldsymbol{Z}_1).$$

## C.1 Regularity Conditions

Condition 1 (Compactness): The true parameter value $\xi_0$ lies in the interior of a known compact set in $\mathbb{R}^p$, where $p$ is the dimension of the parameter space for the regression coefficients.

Condition 2 (Smoothness): The true cumulative baseline hazard functions $\Lambda_{01}(\cdot)$ and $\Lambda_{02}(\cdot)$ are continuously differentiable with strictly positive derivatives within the union of the supports of the monitoring times, denoted by $(0, \tau_1)$ and $(0, \tau_2)$, respectively.

Condition 3 (Bounded covariates): The time-independent covariate vectors $\boldsymbol{Z}_1$ and $\boldsymbol{Z}_2$ are bounded almost surely.

Condition 4 (Monitoring schedule and density smoothness): The number of monitoring visits for $T_1$ and $T_2$, denoted by $K_1$ and $K_2$, respectively, satisfy $E(K_j) < \infty$ for $j = 1, 2$. The conditional density of adjacent monitoring times, $(U_l, U_{l+1})$, for $T_2$, given any $T_1 \in (0, \tau_1)$ and covariates $\boldsymbol{Z}_2$, has continuous second-order partial derivatives with respect to $U_l$ and $U_{l+1}$. Additionally, there exists some constant $\eta > 0$ such that:

$$P\left(\min_l (U_{l+1} - U_l) \geq \eta\right) = 1.$$

Condition 5 (Identifiability): For any two parameter values $(\xi_1, \Lambda_{11}, \Lambda_{21})$ and $(\xi_2, \Lambda_{12}, \Lambda_{22})$, if

$$\int W(u, t_2, \xi_1, \Lambda_{21}) - W(u, t_2, \xi_2, \Lambda_{22}) du = 0$$

with probability 1 for any $t_2 \in (0, \tau_2)$, then it follows that $\xi_1 = \xi_2$, $\Lambda_{11} = \Lambda_{12}$, and $\Lambda_{21} = \Lambda_{22}$.

*Remark 1.* Conditions 1–3 are standard in survival analysis and ensure identifiability and regularity of the likelihood function. Condition 4 ensures that any two adjacent monitoring times for $T_2$ are separated by a positive interval; otherwise, in cases of exact observations, a different treatment would be required. This condition also guarantees the smoothness of the joint density of the monitoring times, following the arguments in [1,2]. Condition 5 ensures parameter identifiability.

## C.2 Proof Outline

The general proof strategy follows similar arguments to [1], [2] and [3]. First, the estimation of the cumulative baseline hazard functions follows the well-established framework of [3] and [4], which show that the maximum likelihood estimator of a monotone cumulative hazard function under interval censoring achieves a cube-root rate of convergence, i.e., $O_p(n^{-1/3})$. Next, the estimation procedure employs a profile likelihood approach, where the cumulative baseline hazard functions are treated as infinite-dimensional nuisance parameters. By profiling out these nuisance parameters, the parametric component $\xi$ can be estimated at the standard $\sqrt{n}$-rate. Finally, the asymptotic normality of $\hat{\xi}$ follows from semi-parametric efficiency theory. Specifically, the efficient information operator remains invertible, and the likelihood function satisfies standard quadratic approximation properties. Moreover, the proof relies on a bundled process approach for handling cumulative hazard functions and covariates, see [1]. As a result, the variance-covariance matrix $\Sigma$ attains the semiparametric efficiency bound.

# APPENDIX D: ADDITIONAL NUMERICAL RESULTS

## D.1 Additional simulation results

### Continuous covariate setting (Study 1 variant).

We present additional simulation results for the independent data setting with a continuous covariate. We considered the following models for $T_1$ and $T_2$, respectively:

$$\lambda_{2i}(t) = \lambda_{20}(t) \exp(\beta_1 T_{1i} + \beta_2 X_i), i = 1, ..., N \tag{5}$$

$$\lambda_{1i}(t) = \lambda_{10}(t) \exp(\gamma X_i), i = 1, ..., N \tag{6}$$

where $X_i \sim \mathrm{N}(0, 1)$ was a continuous covariate, and $(\beta_1, \beta_2, \gamma) = (0.3, 1.0, -0.5)$. The monitoring schedules were generated with the same level of frequency as that for the main paper's independent data setting. The simulation results are presented in Table

1. The performance of the proposed method with a continuous covariate was similar to that with a binary covariate presented in the main paper.

**Study 3B: Robustness to violation of the proportional hazards assumption for $T_1$.**

Table 3 presents the detailed results for Study 3B, which evaluated robustness when the proportional hazards assumption for $T_1$ is violated. Data were generated under a piecewise proportional hazards model for $T_1$,

$$\lambda_{1i}(t \mid Z_i) = \lambda_{10}(t) \exp\left\{ \gamma^{(1)} Z_i \, \mathbb{I}(t \leq \tau) + \gamma^{(2)} Z_i \, \mathbb{I}(t > \tau) \right\},$$

with $\gamma^{(1)} = \gamma = -0.5$ and $\gamma^{(2)} = 0$, where $\tau$ was set to the marginal median of $T_1$ under $Z = 0$ in the corresponding Study 1 scenario. The data-generating mechanism for $T_2 \mid (T_1, \boldsymbol{Z}_2)$, the baseline hazards, and the monitoring/interval-censoring scheme were identical to those in Study 1. For estimation and inference, we intentionally fit the misspecified working PH model $\lambda_{1i}(t \mid Z_i) = \lambda_{10}(t) \exp(\gamma Z_i)$ together with the original working model for $T_2 \mid (T_1, \boldsymbol{Z}_2)$. Under this setting, approximately 20% of $T_1$ observations were right-censored. For comparison, we also report results from midpoint imputation approach (a), in which $T_1$ is imputed by the midpoint of $(L_1, R_1]$ (or by $L_1$ when $R_1 = \infty$) and an interval-censored Cox PH model is fitted for $T_2$. The proposed approach retained negligible bias and near-nominal coverage for $\beta_1$ in both the low- and high-frequency monitoring scenarios, whereas midpoint imputation approach (a) exhibited larger positive bias and lower coverage probability, under the settings we considered.

**Clustered data setting with varying within-subject dependence.**

Table 4 presents simulation results for the clustered data setting under varying strengths of within-subject dependence ($\rho \in \{0.3, 0.5, 0.7\}$), using the cluster bootstrap ($B = 100$ resamples) for variance estimation. We focused on Scenarios 1.1 (low/low monitoring frequency) and 2.2 (high/high monitoring frequency) at sample sizes $n = 100$ and $n = 200$. All other data-generating components—including the baseline hazards, regression parameters $(\beta_1, \beta_2, \gamma) = (0.3, 1.0, -0.5)$, the episode-count distribution, and the monitoring scheme—were identical to those described in the main paper's clustered data simulation. Across all levels of $\rho$, point estimation of $\beta_1$ remained essentially unbiased. The bootstrap standard error (bASE) tracked the empirical standard error (ESE) closely, and confidence-interval coverage probabilities were near the nominal 95% level across all settings. As expected, the empirical standard error increased modestly with stronger within-subject correlation, reflecting the reduced effective sample size in the presence of stronger dependence. These results complement the sandwich-based inference reported in the main paper (Table 2 for $\rho = 0.5$) and demonstrate that both the sandwich and bootstrap approaches provide reliable inference across a range of within-cluster dependence strengths.

## D.2 Additional real data analysis

In this section, we present the graphical visualizations using imputed $T_1$ and $T_2$ values by plotting the estimated time-dependent coefficients for each covariate. The results are presented in Figure 1. The results did not provide strong evidence suggesting a violation of the assumption.

## References

1. Zeng D, Mao L, Lin D. Maximum likelihood estimation for semiparametric transformation models with interval-censored data. *Biometrika* 2016; 103(2): 253–271.

2. Zeng D, Gao F, Lin D. Maximum likelihood estimation for semiparametric regression models with multivariate interval-censored data. *Biometrika* 2017; 104(3): 505–525.

3. Huang J. Efficient estimation for the proportional hazards model with interval censoring. *The Annals of Statistics* 1996; 24(2): 540–568.

4. Groeneboom P, Wellner JA. Information Bounds and Nonparametric Maximum Likelihood Estimation. In: Switzerland: Birkhäuser Boston. 1992.

**Table 1** Simulation results for the independent data setting (with a continuous covariate). Number of replications 10000.

| | | BIAS | ESE[1] | ASE[1] | CP[1] | BIAS | ESE | ASE | CP |
|---|---|---|---|---|---|---|---|---|---|
| Sample size | | Scenario 1.1 | | | | Scenario 1.2 | | | |
| $n = 100$ | $\beta_1 = 0.3$ | 0.005 | 0.075 | 0.072 | 0.943 | 0.006 | 0.073 | 0.071 | 0.946 |
| | $\beta_2 = 1.0$ | 0.042 | 0.242 | 0.237 | 0.947 | 0.045 | 0.243 | 0.234 | 0.946 |
| | $\gamma = -0.5$ | -0.020 | 0.221 | 0.213 | 0.945 | -0.017 | 0.218 | 0.213 | 0.945 |
| $n = 200$ | $\beta_1 = 0.3$ | -0.009 | 0.153 | 0.149 | 0.946 | -0.010 | 0.149 | 0.149 | 0.953 |
| | $\beta_2 = 1.0$ | 0.023 | 0.166 | 0.165 | 0.949 | 0.022 | 0.166 | 0.162 | 0.945 |
| | $\gamma = -0.5$ | -0.001 | 0.051 | 0.049 | 0.944 | -0.001 | 0.050 | 0.049 | 0.946 |
| $n = 400$ | $\beta_1 = 0.3$ | -0.005 | 0.106 | 0.104 | 0.947 | -0.006 | 0.106 | 0.105 | 0.948 |
| | $\beta_2 = 1.0$ | 0.011 | 0.116 | 0.115 | 0.947 | 0.012 | 0.115 | 0.114 | 0.948 |
| | $\gamma = -0.5$ | -0.005 | 0.035 | 0.034 | 0.945 | -0.005 | 0.035 | 0.034 | 0.940 |
| | | Scenario 2.1 | | | | Scenario 2.2 | | | |
| $n = 100$ | $\beta_1 = 0.3$ | 0.011 | 0.074 | 0.072 | 0.946 | 0.012 | 0.074 | 0.071 | 0.942 |
| | $\beta_2 = 1.0$ | 0.040 | 0.248 | 0.237 | 0.942 | 0.039 | 0.243 | 0.234 | 0.944 |
| | $\gamma = -0.5$ | -0.017 | 0.220 | 0.211 | 0.939 | -0.017 | 0.220 | 0.211 | 0.943 |
| $n = 200$ | $\beta_1 = 0.3$ | 0.005 | 0.051 | 0.050 | 0.945 | 0.005 | 0.050 | 0.049 | 0.945 |
| | $\beta_2 = 1.0$ | 0.023 | 0.166 | 0.165 | 0.950 | 0.023 | 0.164 | 0.163 | 0.947 |
| | $\gamma = -0.5$ | -0.008 | 0.151 | 0.148 | 0.948 | -0.009 | 0.148 | 0.148 | 0.950 |
| $n = 400$ | $\beta_1 = 0.3$ | 0.001 | 0.035 | 0.035 | 0.950 | 0.002 | 0.035 | 0.034 | 0.945 |
| | $\beta_2 = 1.0$ | 0.010 | 0.116 | 0.115 | 0.950 | 0.010 | 0.114 | 0.114 | 0.951 |
| | $\gamma = -0.5$ | -0.006 | 0.106 | 0.104 | 0.946 | -0.005 | 0.106 | 0.104 | 0.945 |

[1]: ESE=Empirical standard deviation;
ASE=Average standard error (ASE);
CP=Coverage probability

**Table 2** Interval-censoring (IC) rate, right-censoring (RC) rate, and mean bracket width for $T_1$ and $T_2$ in each simulation study setting. Monitoring Level 1 (low frequency): $c = 1/15$, $d = 1.0$, $K = 30$; Level 2 (high frequency): $c = 19/590$, $d = 0.5$, $K = 60$. Scenario $a.b$: $T_1$ monitored at Level $a$, $T_2$ monitored at Level $b$. In the clustered setting, approximately 25.8% of episodes have $T_2$ unobserved ($O_{2ij} = 0$), which is distinct from right-censoring. The IC/RC rates reported here are among episodes with $T_2$ observed.

| | | $T_1$ | | | $T_2$ | | |
|---|---|---|---|---|---|---|---|
| Study | Scenario | IC% | RC% | Width[1] | IC% | RC% | Width[1] |
| *Study 1:* $\Lambda_1^{(0)}(t) = (t/3)^2$, $\Lambda_2^{(0)}(t) = (t/10)^5$, $(\beta_1, \beta_2, \gamma) = (0.3, 1.0, -0.5)$ | | | | | | | |
| | 1.1 | 100.0 | 0.0 | 0.71 | 100.0 | 0.0 | 0.71 |
| | 1.2 | 100.0 | 0.0 | 0.71 | 100.0 | 0.0 | 0.36 |
| | 2.1 | 100.0 | 0.0 | 0.36 | 100.0 | 0.0 | 0.71 |
| | 2.2 | 100.0 | 0.0 | 0.36 | 100.0 | 0.0 | 0.36 |
| *Study 2:* $\Lambda_1^{(0)}(t) = (t/15)^2$, $\Lambda_2^{(0)}(t) = (t/10)^2$, $(\beta_1, \beta_2, \gamma) = (0.3, 1.0, 0)$ | | | | | | | |
| | 1.1 | 71.6 | 28.4 | 0.72 | >99.9 | <0.1 | 0.68 |
| | 1.2 | 71.5 | 28.5 | 0.72 | >99.9 | <0.1 | 0.35 |
| | 2.1 | 71.7 | 28.3 | 0.36 | >99.9 | <0.1 | 0.68 |
| | 2.2 | 71.7 | 28.4 | 0.36 | >99.9 | <0.1 | 0.35 |
| *Study 3A: same data-generating process as Study 1; Scenarios 1.1 and 2.2 only* | | | | | | | |
| | 1.1 | 100.0 | 0.0 | 0.71 | 100.0 | 0.0 | 0.71 |
| | 2.2 | 100.0 | 0.0 | 0.36 | 100.0 | 0.0 | 0.36 |
| *Study 3B: piecewise-PH for* $T_1$*; same baselines and monitoring as Study 1; Scenarios 1.1 and 2.2 only* | | | | | | | |
| | 1.1 | 80.5 | 19.5 | 1.08 | 99.7 | 0.3 | 1.16 |
| | 2.2 | 80.4 | 19.6 | 0.35 | 100.0 | 0.0 | 0.36 |
| *Clustered: same baselines as Study 1; cluster sizes* $\{1, 2, 3\}$, $P = \{0.3, 0.5, 0.2\}$ | | | | | | | |
| | 1.1 | 100.0 | 0.0 | 0.71 | 100.0 | 0.0 | 0.71 |
| | 1.2 | 100.0 | 0.0 | 0.71 | 100.0 | 0.0 | 0.36 |
| | 2.1 | 100.0 | 0.0 | 0.36 | 100.0 | 0.0 | 0.71 |
| | 2.2 | 100.0 | 0.0 | 0.36 | 100.0 | 0.0 | 0.36 |

[1]Width: mean bracket width ($R_s - L_s$) among interval-censored observations.

**Table 3** Simulation Study 3B results comparing the proposed approach and midpoint imputation (T1-only). Number of replications: 1000.

| | | BIAS | ESE[1] | ASE[1] | CP[1] | BIAS | ESE | ASE | CP |
|---|---|---|---|---|---|---|---|---|---|
| Method | Parameter | Scenario 1.1 (low/low) | | | | Scenario 2.2 (high/high) | | | |
| Proposed | $\beta_1 = 0.3$ | -0.015 | 0.101 | 0.095 | 0.921 | 0.004 | 0.092 | 0.090 | 0.945 |
| Proposed | $\beta_2 = 1.0$ | 0.023 | 0.185 | 0.186 | 0.954 | 0.028 | 0.181 | 0.180 | 0.947 |
| Midpoint (T1-only) | $\beta_1 = 0.3$ | 0.046 | 0.079 | 0.076 | 0.896 | 0.058 | 0.073 | 0.074 | 0.882 |
| Midpoint (T1-only) | $\beta_2 = 1.0$ | 0.017 | 0.167 | 0.170 | 0.956 | 0.009 | 0.161 | 0.163 | 0.949 |

[1]: ESE = Empirical standard deviation; ASE = Average standard error; CP = Coverage probability.

**Table 4** Simulation results for $\beta_1$ in the clustered data setting (Scenarios 1.1 and 2.2, $\rho \in \{0.3, 0.5, 0.7\}$). Number of replications: 1000.

| | | Scenario 1.1 | | | | Scenario 2.2 | | | |
|---|---|---|---|---|---|---|---|---|---|
| Sample size | $\rho$ | BIAS | ESE[1] | bASE[1] | CP[1] | BIAS | ESE | bASE | CP |
| $n = 100$ | 0.3 | −0.001 | 0.054 | 0.053 | 0.938 | 0.005 | 0.054 | 0.053 | 0.944 |
| | 0.5 | −0.000 | 0.058 | 0.057 | 0.943 | 0.007 | 0.058 | 0.057 | 0.937 |
| | 0.7 | 0.001 | 0.063 | 0.063 | 0.943 | 0.008 | 0.064 | 0.063 | 0.938 |
| $n = 200$ | 0.3 | −0.004 | 0.039 | 0.036 | 0.926 | 0.001 | 0.038 | 0.036 | 0.934 |
| | 0.5 | −0.004 | 0.041 | 0.039 | 0.936 | 0.002 | 0.041 | 0.039 | 0.930 |
| | 0.7 | −0.003 | 0.045 | 0.043 | 0.938 | 0.003 | 0.045 | 0.043 | 0.930 |

[1]: ESE = Empirical standard deviation; bASE = Average bootstrap standard error;
CP = Coverage probability

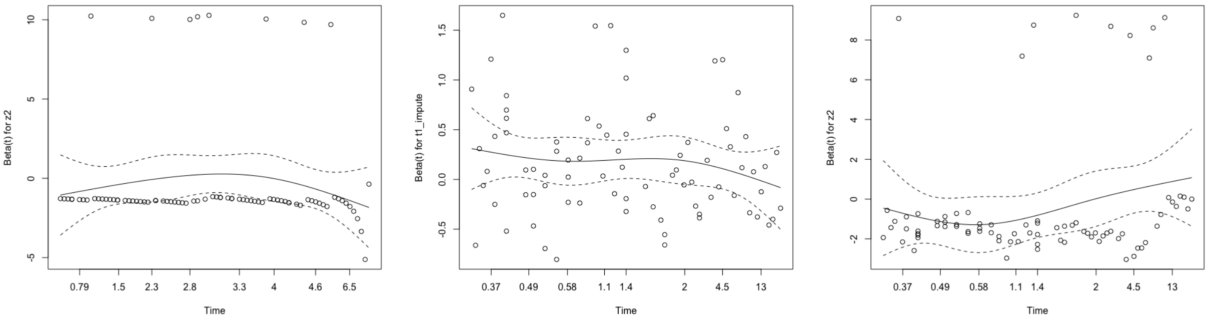


**(a)** Time-dependent coefficient $\beta(t_1)$ estimate for the covariate gender. **(b)** Time-dependent coefficient $\beta(t_2)$ estimate for the imputed covariate $T_1$. **(c)** Time-dependent coefficient $\beta(t_2)$ estimate for the covariate gender.

**Figure 1** Time-dependent coefficient estimates for the model for $T_1$ (Plot (a)) and the model for $T_2$ (Plot (b) and Plot (c)), using midpoint-imputed $T_1$ and $T_2$ values. Plot (a) shows the time-dependent coefficient $\beta(t_1)$ estimate for gender in the model for $T_1$. Plot (b) shows the time-dependent coefficient $\beta(t_2)$ estimate for the imputed covariate $T_1$ in the model for $T_2$. Plot (c) shows the time-dependent coefficient $\beta(t_2)$ estimate for gender in the model for $T_2$.
